# Bioelectricity harvesting from microorganism: review of recent advancements in utilizing the bioelectric properties of fungi for powering small-scale robotic systems

**DOI:** 10.3389/ffunb.2025.1739847

**Published:** 2026-01-15

**Authors:** Rice Ul Islam, Ayite Ayele Kossiwa Chantal, Ariful Islam, Okpalanwabude Stanley Somtochukwu, Ritu Raj Poudyal, Cashline Bless Wesseh, Omar Ibnul Faiyaz, Xiangzeng Kong, Xuan Wei

**Affiliations:** 1 College of Mechanical and Electrical Engineering, Fujian Agriculture and Forestry University, Fuzhou, Fujian, China; 2 Institute of Artificial Intelligence for Agriculture and Forestry, Fuzhou, Fujian, China; 3 College of Plant Protection, Fujian Agriculture and Forestry University, Fuzhou, Fujian, China; 4 Department of Mechanical Engineering, University of Windsor, Windsor, ON, Canada

**Keywords:** bioelectricity, biohybrid robotics, extracellular electron transfer, fungal mycelium, microbial fuel cells

## Abstract

The growing need for sustainable energy sources has led to the exploration of bioelectricity generation from microorganisms, with fungi showing considerable potential for powering small-scale robotic systems. Fungal bioelectricity stems from the ability of fungal mycelium to facilitate extracellular electron transfer, a process that can be exploited in microbial fuel cells (MFCs) for clean energy production. This field is gaining traction as fungi, with their extensive mycelial networks, offer unique conductive properties. These networks, providing a large surface area and excellent conductivity, make fungi well-suited for incorporation into fungal-based microbial fuel cells (FMFCs). Successful FMFC design and optimization require attention to critical factors such as electrode material, microbial interactions, and environmental conditions to enhance performance. Moreover, the use of fungi in small-scale robotic systems, forming biohybrid robots, holds significant promise for autonomous operations in applications like environmental monitoring and bio-inspired robotics. While fungal bioelectricity presents exciting opportunities, challenges such as energy efficiency, scalability, and integration persist. Nevertheless, ongoing research continues to advance the development of self-sustaining, environmentally friendly robotic systems powered by fungal bioelectricity, providing new avenues in renewable energy and robotics.

## 1 Introduction

In the contemporary landscape of intelligent automation and sustainable innovation, the advancement of autonomous and miniaturized systems, including microrobots, biohybrid platforms, and embedded environmental sensors, is reshaping diverse domains such as environmental monitoring, precision agriculture, biomedical diagnostics, and soft robotics. A central challenge hindering the real-world deployment of these microsystems is the absence of a compatible, long-lasting, and renewable power source that meets their spatial and functional constraints. Conventional energy storage technologies, such as lithium-ion batteries and micro-capacitors, though efficient in macro-scale applications, often fall short in fulfilling the requirements of flexibility, biocompatibility, environmental safety, and energy self-sufficiency critical for microscale robotic and sensing platforms (Riaz et al., 2021). These systems demand energy solutions that are lightweight, inherently safe, and capable of harvesting energy from ambient sources to enable prolonged, autonomous operation. The growing urgency of climate change, global warming, and fossil fuel depletion necessitates a paradigm shift toward non-depletable, environmentally sustainable energy alternatives.

In this context, bioelectrochemical systems (BESs) have emerged as promising platforms capable of converting biochemical energy into electrical power, driven by the metabolic activity of microorganisms (Naradasu et al., 2020). These systems utilize microbes to oxidize organic substrates, releasing electrons that are transferred to an external circuit via direct or mediated extracellular electron transfer (EET), thereby generating a continuous electric current. BESs offer several advantages, including sustainability, operation under ambient conditions, and the ability to utilize biodegradable or waste-derived substrates, positioning them as ecologically favorable alternatives for low-power energy generation (Sleutels et al., 2012). Various BES configurations have been developed, such as microbial desalination cells (MDCs) (Jatoi et al., 2022), microbial electrosynthesis cells (MECs) (Tanguay-Rioux et al., 2023), enzymatic biofuel cells (EBCs) (Li et al., 2023), electrolysis cells (ECs) (Zhang et al., 2023), microbial solar cells (MSCs) (Pant et al., 2012), biobatteries (Apollon et al., 2022), constructed wetland microbial fuel cells (CW-MFCs) (Ji et al., 2023), and microbial fuel cells (MFCs). Among these, MFCs are the most established, with the earliest known example developed in 1911 by Potter using *Escherichia coli*, generating a current of 0.2 mA (M. C. Potter, 1911). Subsequent advancements, including Cohen’s (1931) design of microbial half-fuel cells in series yielding ~35 V at 2 mA, (Cohen, 1931) and the work of Sisler (1961, 1962), Davis and Yarbrough (1962), and Davis (1963), contributed to the foundational development of microbial electricity generation (Sisler, 1961; Davis and Yarbrough, 1962; Sisler, 1962; Davis, 1963). Renewed interest in the early 1990s, catalyzed by the work of Allen and Bennetto (1993), led to substantial progress in increasing power density and reducing material and operational costs. Afroz and his colleagues categorized MFCs as either laboratory-scale or *in situ* systems, with *in situ* configurations further differentiated into docked, floating, or submerged aquatic systems (Afroz et al., 2021). Terrestrial MFCs encompass various architectures, including single- or dual-chambered, up-flow, stacked, tubular, and forced-flow designs (Ramya and Senthil Kumar, 2022). Extensive research has focused on the utilization of diverse microorganisms, particularly electrogenic bacteria such as *Geobacter sulfurreducens* (Lovley et al., 2011), *Shewanella oneidensis* (Beliaev et al., 2008), and *Pseudomonas aeruginosa* (Ali et al., 2018), owing to their well-characterized EET mechanisms and high electrogenic potential. These bacteria have demonstrated efficacy in powering environmental sensors, low-power electronics, and biohybrid robotic systems under controlled conditions, enabling rudimentary functionalities including wireless communication, locomotion, and environmental responsiveness.

However, bacterial MFCs face significant limitations. These include limited substrate specificity, short biofilm longevity, high material costs, susceptibility to environmental perturbations, and relatively low power outputs in non-laboratory settings (Sarma and Kakati, 2022). Moreover, the integration of bacterial MFCs into heterogeneous matrices such as soil or biological tissues is impeded by factors such as poor adhesion, structural fragility, and the requirement for continuous microbial culture maintenance. These challenges have led researchers to explore alternative microbial domains, including fungi, which offer potential advantages in resilience, metabolic diversity, and biofilm stability, Historically, fungal-based MFCs received limited attention due to early assumptions about their weak electrogenic capabilities and lower power yields compared to bacterial systems (Potter, 1911). However, recent advancements have revealed that fungi possess unique bioelectrochemical properties that are increasingly being recognized for their potential in sustainable energy generation (Sarma et al., 2021; Umar et al., 2023). Fungi, as eukaryotic organisms, demonstrate a high capacity to degrade complex organic matter such as lignin and cellulose, and can thrive in diverse and extreme environments. Their filamentous hyphal structures support dense, stable and highly adherent mycelial biofilms, which facilitate efficient electron transfer over prolonged durations. In addition to their structural advantages, many fungi secrete redox-active metabolites such as melanin, flavins, phenolics and quinones that function as natural electron shuttles between the fungal cells and the electrode surfaces. Notably, species such as *Aspergillus niger*, *Phanerochaete chrysosporium, Schizophyllum commune*, *Ganoderma lucidum*, *Trametes pubescens* and *Trametes versicolor* have demonstrated measurable current generation either independently or in synergistic microbial consortia. These attributes make fungi not only viable but also potentially superior candidates for integration into flexible, decentralized bioelectric systems. A noteworthy recent development in fungal bioelectrochemistry is the creation of 3D-printed, cellulose-based fungal batteries (Reyes et al., 2024). This work demonstrated that biodegradable, additively manufactured electrodes can be successfully combined with electrogenic fungi, offering a new direction for bioelectrode design. The study also presented a dual-fungus MFC setup and, importantly, documented the electrogenic behavior of *Trametes pubescens*, a white-rot basidiomycete not previously examined in this context. The combination of ligninolytic fungal species with structured 3D-printed electrodes led to stronger mycelial attachment and higher electrical output, underscoring the potential of these hybrid systems for advancing fungal-based energy technologies. Moreover, edible and medicinal macrofungi, such as *Ganoderma lucidum* and *Schizophyllum commune* which are commonly used in functional food and nutraceutical industries, are gaining interest for their dual benefits of safety and scalability. Their biofilm-forming capabilities and production of oxidative enzymes make them suitable for applications at both the anode and cathode (Sharma et al., 2024; Wu et al., 2025). This opens up exciting possibilities for developing biocompatible power sources for medical devices, ingestible electronics and biodegradable environmental sensors.

Despite these promising attributes, fungal electrogenesis has received significantly less attention than bacterial counterparts. Recent findings, however, suggest that fungi possess several unique mechanisms for electron generation and transfer, including transmembrane ion fluxes, biosynthesis of extracellular redox compounds, and direct or mediated extracellular electron transfer. These mechanisms, which differ markedly from those in prokaryotic systems, will be examined in detail in the subsequent sections. This review provides a comprehensive synthesis of fungal-based electricity generation, with an emphasis on the mechanistic basis of fungal electrogenesis, the potential of both micro- and macrofungi and their applicability in sustainable bioelectrochemical technologies.

## 2 Microorganisms in bioelectricity generation

**Figure 1:**
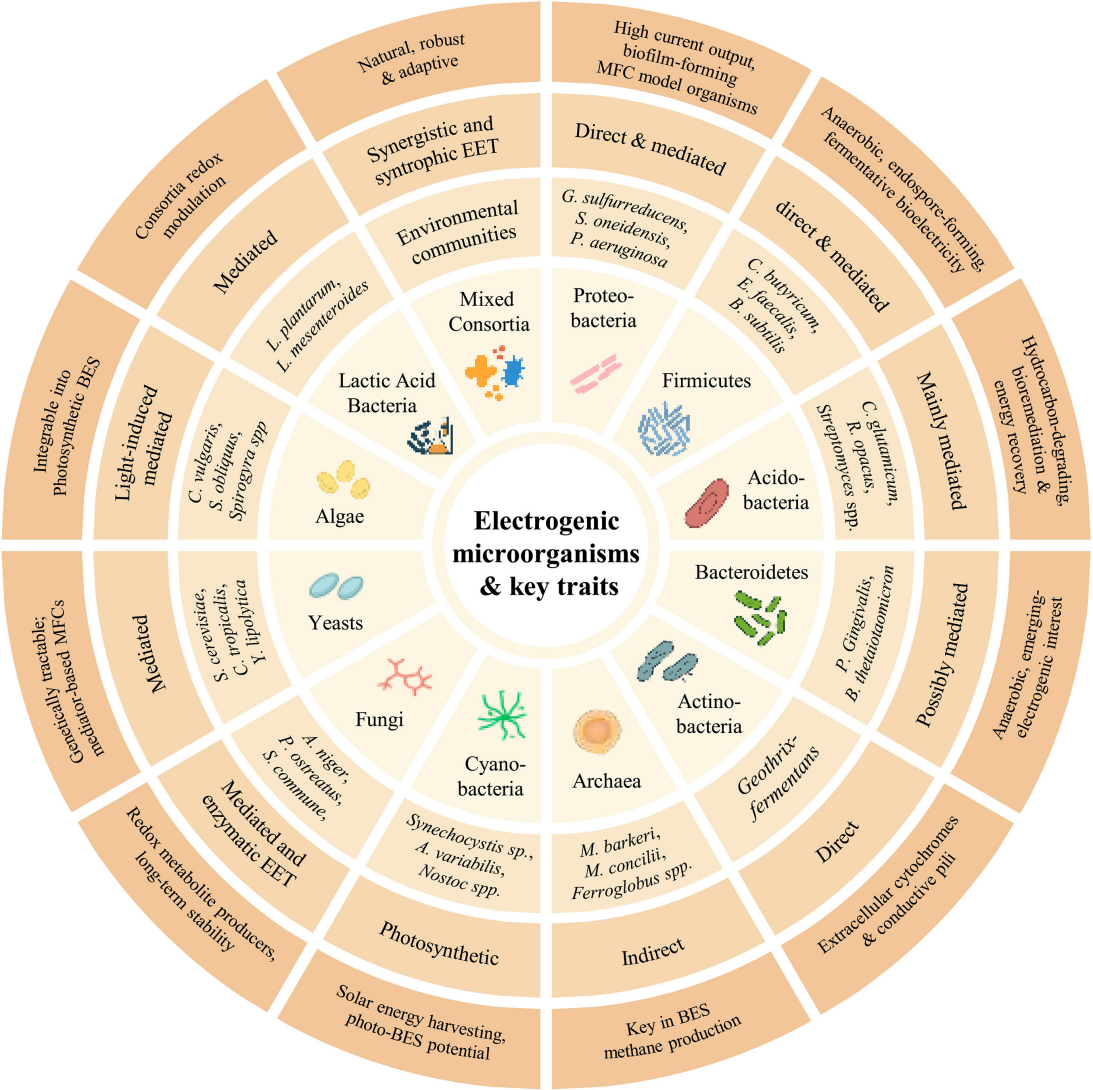
Circular infographic illustrating major classes of electrogenic microorganisms, their electron transfer mechanisms (direct, mediated, enzymatic, or photosynthetic), and bio electrochemical applications such as microbial fuel cells, methane production, and solar-driven systems.

The pursuit of sustainable energy alternatives has led to significant interest in unconventional sources such as microorganisms for bioelectricity generation. Traditional energy production initially relied on first-generation sources, such as food and oil crops. However, these sources pose critical limitations, including extensive land requirements, competition with food supply, and relatively lower energy yields compared to microbial systems (Enamala et al., 2018). In contrast, microbial fuel cells can utilize food waste, kitchen residues, and agricultural by-products as substrates, enabling simultaneous waste valorization and electricity production, which significantly improves sustainability and reduces dependency on edible crops (Oliveira et al., 2013). Third-generation bioenergy technologies, utilizing microorganisms as biofactories for energy production have emerged (Enamala et al., 2018; Pisciotta and Blessing, 2022). Microorganisms offer several advantages, including faster growth rates and minimal spatial demands, thereby offering a scalable and efficient alternative to crop-based bioenergy systems. One of the most promising innovations in this domain is the microbial fuel cell (MFC), a bioelectrochemical system capable of generating electricity through the metabolic activities of microorganisms, specifically via the hydrolysis and fermentation of biomolecules (Apollon, 2023). The microbial ability to generate bioelectricity has attracted considerable scientific attention due to its potential in diverse applications such as renewable energy production, wastewater treatment, biosensing, and environmental remediation. Central to this phenomenon is microbial bioelectrochemistry, an interdisciplinary field that investigates the electrochemical interactions between microbial metabolism and solid-state electron acceptors within bioelectrochemical systems (BESs) (Hassan et al., 2021). BESs harness the metabolic capacity of electrogenic or electroactive microorganisms, which can transfer electrons generated from the oxidation of organic or inorganic substrates directly to an electrode (Naradasu et al., 2020). This process is facilitated through two principal extracellular electron transfer (EET) mechanisms: direct electron transfer (DET) and mediated electron transfer (MET) (Zhao et al., 2021). In DET, microorganisms convey electrons to electrode surfaces through outer membrane redox-active proteins such as c-type cytochromes, or conductive pili, commonly referred to as microbial nanowires (Vargas et al., 2013). In contrast, MET employs soluble redox mediators that act as electron shuttles between the microbial cell and the electrode. These mediators may be endogenously synthesized by the microbes or externally introduced to extend the reach of electron transfer in scenarios where direct physical contact is not feasible (Marsili et al., 2008; Thapa et al., 2022). Historically, bacterial species have been the focus of research in microbial bioelectrochemistry due to their high EET efficiency, well-characterized metabolic pathways, and ease of genetic manipulation (Sreelekshmy, 2020; Garbini et al., 2023). Nonetheless, emerging evidence suggests that other microbial groups also exhibit electrogenic capabilities. These include archaea (Wang et al., 2020), microalgae (Saratale et al., 2017), fungi (Umar et al., 2023), and yeasts (Sayed and Abdelkareem, 2017), particularly under specialized or extreme environmental conditions (**Table 1**, **Figure 1**). Such discoveries have significantly expanded the phylogenetic and functional scope of electrogenic microorganisms, presenting new opportunities for advancing fundamental understanding and applied development of bioelectrochemical technologies.

**Table 1:** Classes of electrogenic microorganisms and their key characteristics.

Class/group	Representative genera/species	Electron transfer mechanism	Notable features	Reference(s)
Proteobacteria (Gram-negative)	*Geobacter sulfurreducens*, *Shewanella oneidensis*, *Shewanella* ANA-3, *Pseudomonas aeruginosa*	Direct and mediated electron transfer (cytochromes, nanowires, flavins)	High current output, biofilm-forming, model organisms for microbial fuel cells (MFCs)	(Reyes et al., 2012; Hu et al., 2021; Zhang et al., 2024; Yin et al., 2025)
Firmicutes (Gram-positive)	*Clostridium butyricum*, *Enterococcus faecalis*, *Bacillus subtilis*	Mixed (direct and mediated) electron transfer	Anaerobic growth, endospore-forming, potential for fermentative bioelectricity	(Kumar et al., 2017; Ryu and Choi, 2021; Parihar et al., 2022)
Actinobacteria	*Corynebacterium glutamicum*, *Rhodococcus opacus*, *Streptomyces* spp.	Mainly mediated electron transfer	Capable of degrading complex hydrocarbons, with potential in bioremediation and energy recovery	(Lee et al., 2019b; Balagurunathan et al., 2020)
Bacteroidetes	*Bacteroides thetaiotaomicron*, *Porphyromonas gingivalis*	Possibly mediated	Abundant in anaerobic systems, emerging interest in electrogenesis	(Zou et al., 2022; Garimella et al., 2024)
Acidobacteria	*Geothrix fermentans*	Direct electron transfer	Produces extracellular cytochromes and conductive pili	(Bond and Lovley, 2003; Mehta-Kolte and Bond, 2012)
Archaea (Methanogens)	*Methanosarcina barkeri*, *Methanosaeta concilii*, *Ferroglobus* spp.	Indirect (via methane metabolism and syntrophic associations)	Key players in bioelectrochemical methane production (BES systems)	(Gao and Lu, 2021; Yu et al., 2021)
Cyanobacteria (Phototrophs)	*Synechocystis* sp., *Anabaena variabilis*, *Nostoc* spp.	Photosynthetic electron transfer	Can harvest solar energy; potential for photo-bioelectrochemical systems	(Tanaka et al., 1985; Sawa et al., 2017)
Fungi (Filamentous)	*Aspergillus niger*, *Pleurotus ostreatus*, *Schizophyllum commune*, *Trichoderma* spp.*, Trametes pubescens*	Mediated and enzymatic EET	Produce redox-active metabolites (e.g., melanin, phenazines); durable and stable in long-term operations.	(Umar et al., 2023; Reyes et al., 2024)
Yeasts (Unicellular fungi)	*Saccharomyces cerevisiae*, *Candida tropicalis*, *Yarrowia lipolytica*	Mediated electron transfer	Easy to manipulate genetically; useful in mediator-based MFCs	(Sayed and Abdelkareem, 2017)
Algae (Eukaryotic phototrophs)	*Chlorella vulgaris*, *Scenedesmus obliquus*, *Spirogyra* spp.	Light-induced mediated transfer	Can be integrated into photosynthetic bioelectric systems	(Da Costa and Hadiyanto, 2018; Mekuto et al., 2020)
Lactic Acid Bacteria (LAB)	*Lactobacillus plantarum*, *Leuconostoc mesenteroides*	Mediated electron transfer	Can modulate redox reactions in microbial consortia; potential for co-culture systems.	(Freguia et al., 2009; Pham et al., 2022)
Mixed Consortia	Environmental communities from soil, sludge, and wastewater	Synergistic and syntrophic EET	Naturally occurring systems with robust, adaptive electron transfer capabilities	(Choudhary et al., 2024; Mirza et al., 2025)

### 2.1 Bacteria

Electrogenic bacteria constitute the most comprehensively investigated group within the field of microbial bioelectrochemistry. The earliest documented instance of electricity generation by microorganism’s dates back over a century, when Potter (1911) reported the development of an electric potential by *Bacillus coli (Escherichia coli)* and *Saccharomyces cerevisiae* cultured in a galvanic cell. By immersing platinum electrodes in a medium containing bacterial and yeast suspensions supplemented with glucose, Potter observed a voltage range of 0.3–0.5 V. Although this observation provided foundational insights, the underlying mechanisms of microbial electricity generation were not elucidated and thus attracted limited scientific attention at the time. Subsequently, Cohen (1931) demonstrated that connecting multiple microbial fuel cells (MFCs) in series could produce voltages up to 35 V, further underscoring the electrochemical potential of microbial systems (Cohen, 1931). These pioneering investigations laid the groundwork for the emergence of electro microbiology; a novel interdisciplinary discipline concerned with electron exchange processes between microorganisms and electronic interfaces (Lovley, 2012). This scientific evolution eventually culminated in the conceptualization and development of microbial fuel cells (Allen and Bennetto, 1993), designed to harness microbial metabolic processes for direct electricity generation. The discovery of *Shewanella oneidensis* MR-1 marked a pivotal advancement in the field. The earliest identification of this organism, then known as *Alteromonas putrefaciens* MR-1, was reported by Myers and Nealson (1988), who demonstrated its capacity for manganese reduction and growth with manganese oxide as the sole electron acceptor. The taxonomic reclassification that recognized the organism as *Shewanella* was later refined through molecular and phylogenetic analyses. Subsequently, Kim et al. (1999) provided important advancements by characterizing the species under its modern nomenclature, *Shewanella oneidensis*. This facultative anaerobic, Fe(III)-reducing bacterium was the first to be recognized for its electrogenic capabilities (Ikeda et al., 2021). Building on this, Bond and Lovley (2003) identified *Geobacter sulfurreducens*, a species capable of significantly enhancing MFC performance through efficient electron transfer mechanisms (Bond and Lovley, 2003). *Geobacter sulfurreducens* strains PCA and KN400, along with *Shewanella oneidensis* MR-1, have since become model organisms in microbial electrochemistry (Koch and Harnisch, 2016). *Geobacter* species are characterized by their ability to perform direct electron transfer (DET) using conductive pili (nanowires) and outer membrane-bound c-type cytochromes, enabling electron flow to insoluble metal oxides and electrodes. These features contribute to their high current output in MFC configurations (Reguera and Kashefi, 2019). In contrast, *Shewanella* species exhibit a broader metabolic versatility, functioning as facultative anaerobes capable of employing both DET and mediated electron transfer (MET) (Liu et al., 2025). The MET pathway in *Shewanella oneidensis* is notably facilitated by the endogenous production and secretion of flavins, which act as soluble redox mediators that enhance electron transfer between the microbial cell and the electrode interface, a process that critically depends on the Mtr respiratory pathway (Coursolle et al., 2010). Both *Geobacter* and *Shewanella* species are well-adapted to anoxic environments and demonstrate the ability to metabolize diverse organic substrates. These attributes not only position them as model organisms in microbial electrochemical systems but also render them highly suitable for applications in sustainable energy production, environmental remediation, and biotechnological processes involving bioelectrochemical transformations. Beyond *Geobacter* and *Shewanella*, several other bacterial genera, including *Desulfuromonas, Clostridium*, and *Pseudomonas*, have been explored for their electrogenic potential (Garbini et al., 2023). However, these species typically exhibit lower electron transfer rates and current densities, making them less favorable for high-performance MFC applications.

### 2.2 Microalgae

Microalgae are unicellular, photosynthetic microorganisms increasingly recognized for their potential in bioelectricity generation through algae-assisted microbial fuel cells (MA-MFC). Unlike traditional electroactive bacteria, microalgae possess unique advantages: they do not only generate bioelectricity but also function as natural oxygenators in the cathodic chamber and contribute to CO_2_ fixation, enhancing system sustainability (Kruzic and Kreissl, 2009; Da Costa and Hadiyanto, 2018).

In MFC systems, microalgae can play dual roles:

1. Anodic role: certain species such as *Chlorella pyrenoidosa* and *Nitzschia palea* are capable of directly donating electrons to the anode via photosynthetically generated reducing equivalents, effectively substituting traditional electroactive microorganisms (EAMs) (Xu et al., 2015; Khan et al., 2022)
2. Cathodic role: photosynthetic oxygen evolution from live algal cultures enhances the cathodic reduction of oxygen, eliminating the need for costly aeration systems or chemical oxidants (Shukla and Kumar, 2018)

Microalgae harness light and water to fix atmospheric CO_2_ through the fundamental photosynthetic process (Equation 1), producing biomass while releasing oxygen as a byproduct. The generated oxygen, plays a pivotal role in sustaining the cathodic half-reaction, closing the redox loop and facilitating continuous current generation (Kruzic and Kreissl, 2009).


(1) $${\text{Photosynthesis}\;\text{Reaction}\text{:}\text{ CO}}_{2}+{\text{H}}_{2}\text{O}+\text{Light}\to {\text{CH}}_{2}\text{O}+{\text{O}}_{2}$$

Notably, microalgae have been successfully integrated into photobioreactor-assisted MFCs to achieve stable long term bioelectric output. For example, Strik et al. (2011) reported a continuous power output of 539 mA m-2 over five months using a coupled photobioreactor-MFC system (Strik et al., 2011). These results underscore the viability of microalgae as functional components of bio electrochemical systems, contributing both bioelectricity and auxiliary environmental services such as CO2 sequestration and wastewater remediation (Mekuto et al., 2020). Further enhancements have been emerged through innovations in electrode surface modification (Elmekawy et al., 2014), mixed microbial consortia integrating bacteria and algae (Cui et al., 2014), and optimized light regimes and nutrient conditions for maximizing photosynthetic efficiency (Reddy et al., 2019). While numerous studies have emphasized the wide-ranging applications of microalgae, certain challenges persist. The slower electron transfer kinetics in autotrophic algae compared to bacteria, high internal resistance, and dependency on solar input limit performance under real world conditions (Pandit et al., 2012). Moreover, energy losses due to high redox potentials and suboptimal bio-cathode performance remain critical bottlenecks (Mallick et al., 2016). Despite these hurdles, microalgae offer a multifunctional platform for sustainable bioelectricity generation in MFC’s combining low operational costs, bio-oxygenation, waste remediation and carbon neutrality. Further progress hinges on strain engineering, photobioreactor design and electrode-microbe interface optimization to fully realize their bio electrochemical potential.

### 2.3 Archaea

Although traditionally overlooked in the context of bioelectrochemical systems, archaea have recently gained recognition as potent electrogenic microorganisms, particularly due to their inherent resilience in extreme environmental niches such as high salinity, temperature, and acidity. Among them, methanogenic archaea, including species from the genera *Methanosarcina* and *Methanobacterium*, have demonstrated the ability to participate in direct electron uptake processes at the cathode, facilitating the reduction of carbon dioxide to methane through a mechanism known as electromethanogenesis (Gao and Lu, 2021). Species such as *Methanosarcina barkeri* can acquire electrons directly from adjacent microbial populations, thereby engaging in syntrophic associations. Notably, *Methanothrix barkeri* is reported to utilize direct interspecies electron transfer (DIET) pathways to obtain electrons from *Geobacter metallireducens* (Rotaru et al., 2014). Additionally, *Methanosarcina acetivorans* have been shown to carry out anaerobic oxidation of methane while coupling this metabolism to Fe (III) reduction, mediated through extracellular electron transfer (EET) mechanisms (Holmes et al., 2019). Furthermore, *Methanosarcina mazei* is reported to engage in electromethanogenesis under conditions where conventional electron shuttles such as hydrogen or formate are absent (Gao and Lu, 2021). In the context of electron transport, membrane-associated multiheme c-type cytochromes (MHCs) and electrically conductive appendages have been extensively studied in electrogenic bacteria like *Geobacter* and *Shewanella*. While these structures are uncommon in archaea, emerging evidence has identified similar features in certain methanogens, suggesting that archaea may possess unique and potentially more robust EET pathways. This biochemical and structural distinction underscores the potential of methanogenic archaea in applications such as microbial electrosynthesis and biogas upgrading. Their distinctive lipid membrane compositions and redox enzyme machinery present novel opportunities for advancing the understanding and engineering of archaeal electron transfer systems.

### 2.4 Fungi

Fungi have traditionally remained relatively underrepresented in bioelectrochemical systems (BES), but recent advancements underscore their potential as versatile and sustainable biocatalysts in microbial fuel cells (MFCs) (Umar et al., 2023). These eukaryotic organisms exhibit remarkable metabolic flexibility, enabling them to degrade complex organic substrates such as lignocellulosic biomass, agro-industrial residues, and synthetic polymers while liberating electrons that can be harvested as bioelectricity. Unlike bacteria, fungi possess distinct structural and biochemical properties that contribute to their functionality in MFCs. Notably, fungi can be broadly categorized into microfungi (unicellular yeasts and filamentous molds) and macrofungi (multicellular mushrooms and other fruiting body-forming species), both of which play important roles in bioelectricity generation. Microfungi, including genera such as *Aspergillus, Penicillium, Trichoderma, Saccharomyces, Candida*, and *Kluyveromyces*, are widely utilized in fungal-based fuel cells (FFCs). These species facilitate extracellular electron transfer (EET) either directly via cytochrome c-dependent pathways or through secreted redox-active metabolites. They are also capable of thriving under anaerobic or low-oxygen conditions, often without the need for external electron mediators. Yeasts such as *Kluyveromyces marxianus, Candida melibiosica*, and *Saccharomyces cerevisiae* have been shown to produce significant power outputs in MFCs while maintaining a non-pathogenic profile and tolerating a wide range of operational conditions (**Table 2**). These properties make microfungi attractive for applications in integrated wastewater treatment and energy recovery systems.

**Table 2:** Comparative overview of performance parameters in fungal-based microbial fuel cells (FMFCs).

Fungal species	Substrate used	Power density (mW/m²)	Voltage output (V)	Current density (mA/m²)	Electrode material	Type of MFC	Operating conditions	Reference
*Aspergillus niger*	Glucose (15g/L)	Not specified	0.89	145	Carbon felt electrodes	Dual-chamber	Incubation at 35°C for 48 h, glucose-based synthetic medium	(Osman et al., 2018)
*Aspergillus niger*	Sugarcane bagasse	Not specified	0.0013–0.0042	Not specified	Copper and graphite rods	Dual-chamber	15-day observation, ambient conditions in the lab	(Sari Sekar Ningrum et al., 2023)
*Aspergillus* sp.	Polyethylene (Plastic)	1240 ± 60	0.572 ± 0.024	3.608 ± 0.249	Activated Carbon (AC) and Zinc (Zn)	Single-chamber	pH 6.57 ± 0.27, Conductivity 257.12 ± 20.9 mS/cm, 45 days of operation	(Rojas-Flores et al., 2024)
*Galactomyces reessii*	Industrial rubber wastewater	59	0.145	278	Coconut coir	Dual-chamber	Laccase-based biocathode; coconut coir used as support; anaerobic anode chamber; supplemented with sulfate and pH buffer	(Chaijak et al., 2018)
*Phanerochaete chrysosporium*	Wheat Straw Hydrolysate	14.25 mW/m²	0.571 ± 0.024 V	46.97 mA/m² (1st day) to 427.27 mA/m² (10th day)	Bow-shaped carbon fiber (anode), Stainless steel wire (cathode)	Single-chamber	Inoculated with P. fermentans, incubated at 30°C for 12 days	(Shrivastava and Sharma, 2023)
*Phlebia floridensis*	Wheat Straw Hydrolysate	65.09 mW/m²	0.672 ± 0.014 V	115.15 mA/m² (1st day) to 542.42 mA/m² (12th day)	Bow-shaped carbon fiber (anode), Stainless steel wire (cathode)	Single-chamber	Inoculated with P. fermentans, incubated at 30°C for 12 days	(Shrivastava and Sharma, 2023)
*Phlebia brevispora*	Wheat Straw Hydrolysate	50.75 mW/m²	0.594 ± 0.025 V	87.87 mA/m² (1st day) to 478.78 mA/m² (5th day)	Bow-shaped carbon fiber (anode), Stainless steel wire (cathode)	Single-chamber	Inoculated with P. fermentans, incubated at 30°C for 12 days	(Shrivastava and Sharma, 2023)
*Saccharomyces cerevisiae*	Glucose, Swine wastewater	294	0.420	700	Graphite plates (40×70×2 mm)	Dual-chamber	MFC inoculated with pure culture (24 h, 30°C, 100 ml flask, no agitation). Mediated with thionine (500 mM) for increased electrogenic biofilm formation. External resistances varied between 65,535-1 Ω for polarization curves.	(Rahimnejad et al., 2012)
*Saccharomyces cerevisiae*	Glucose	56.0	0.283	198.6	Carbon cloth	Single-chamber	pH 5.5, 30°C, aerobic cathode, external resistance 500 Ω	(Xie et al., 2022)
*Trametes versicolor*	Glucose (cathode-side, oxygen as e- acceptor)	Not specified	Not specified	42.81 ± 4.91	Nylon sponge immobilization	Dual-chamber	Biocathode with immobilized *T. versicolor*, ambient lab conditions	(Ottoni et al., 2023)
*Tametes pubescens*	Cellulose-based 3D-printed conductive hydrogel	31.2	0.39	102.4	3D-printed carbon-based cellulose electrodes (biodegradable)	Dual-fungi MFC (co-culture)	Additively manufactured structured electrodes; ambient temperature; dual-fungi configuration enhancing colonization	Reyes et al. (2024)

Macrofungi, particularly edible mushrooms such as *Ganoderma lucidum*, *Trametes pubescens, T. versicolor, Pleurotus ostreatus, Lentinula edodes, Schizophyllum commune*, and *Psathyrella candolleana*, represent an emerging class of electrogenic organisms. Their filamentous mycelial networks form dense, three-dimensional biofilms that enhance adhesion to electrode surfaces and enable efficient, long-range electron transfer. These fungi produce a variety of extracellular oxidative enzymes, such as laccases and lignin peroxidases, which contribute to the degradation of lignocellulosic and plastic-derived waste while facilitating electron release. Additionally, their production of phenolic compounds and melanin supports mediated electron transfer, further enhancing their utility in BES applications. Edible macrofungi are especially promising due to their safety, scalability, and compatibility with sustainable agricultural practices. Their dual role in bioelectricity generation and bioremediation reinforces their importance in circular bioeconomy strategies. These fungi have been effectively deployed across diverse MFC designs with various electrode materials, including carbon felt, stainless steel wire, activated carbon, and more recently 3D-printed carbon electrodes (**Table 2**). Fungal-based MFCs have demonstrated impressive power densities using various configurations and electrode materials. For example, *Trametes versicolor, Ganoderma lucidum, Galactomyces reessii, Aspergillus* spp.*, Kluyveromyces marxianus*, and *Hansenula anomala*, have demonstrated substantial power densities, reportedly reaching 1200 mW/m^3^, 207 mW/m^3^, 1163 mW/m^3^, 438 mW/m^3^, 850,000 mW/m^3^, and 2900 mW/m^3^, respectively (Umar et al., 2023). These outputs are partly attributed to the structural attributes of fungal hyphae, which facilitate efficient electron transport via expansive conductive networks, enhancing the stability and intensity of power generation in external electrochemical operations. Due to these distinctive properties, fungi, particularly yeasts, are increasingly preferred over bacterial systems for integrated wastewater treatment and bioelectricity production (Sayed and Abdelkareem, 2017). In addition to electricity generation, many fungal species, especially oleaginous members of the Zygomycota phylum, produce fatty acids such as oleic and palmitic acid, making them suitable candidates for biodiesel production. Anaerobic fungi further enhance biogas yield due to their multienzyme complexes that break down biomass into fermentable intermediates. The integration of fungi into BESs provides several ecological and technological advantages over bacterial systems. These include a broader substrate range, higher resistance to environmental stresses, more effective colonization of electrode surfaces, and the ability to function in systems with mild operational conditions. Yeasts, in particular, have demonstrated the capacity to generate electricity from industrial effluents without requiring chemical additives, supporting their role in low-cost, low-maintenance fuel cell systems (Christwardana et al., 2018). Studies by Gunawardena et al. (2008); Schaetzle et al. (2008), and Hubenova and Mitov (2015) affirm the viability of fungal species as alternative biocatalysts in sustainable energy frameworks. Recent advancements, including the 2024 3D-printed fungal battery study (Reyes et al., 2024), further demonstrate the potential of structured, additively manufactured electrodes combined with fungal systems to enhance electrogenic performance.

## 3 Mechanisms of fungal bioelectricity generation

**Figure 2:**
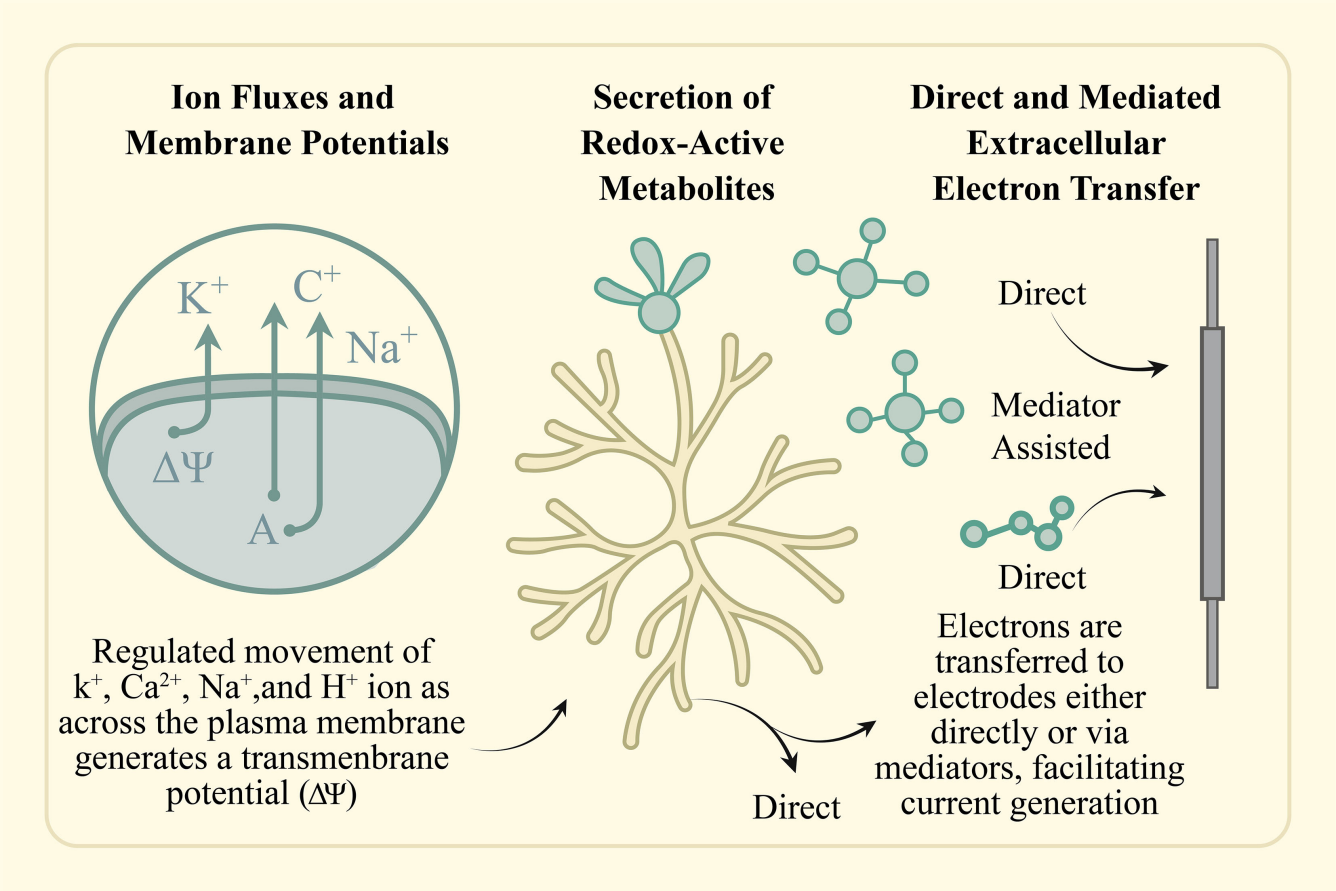
Schematic representation of key mechanisms through which fungi generate bioelectricity: (1) Ion fluxes and membrane potentials—regulated movement of K^+^, Ca²^+^, Na^+^, and H^+^ ions across the plasma membrane generates a transmembrane potential (ΔΨ); (2) Secretion of redox-active metabolites—fungi release extracellular compounds that participate in electron transfer; (3) Direct and mediated extracellular electron transfer (EET)—electrons are transferred to electrodes either directly or via mediators, facilitating current generation in microbial fuel cells.

Fungi exhibit a suite of distinctive bioelectrochemical traits that render them promising biocatalysts for microbial fuel cell (MFC) technologies. In contrast to prokaryotic organisms, fungal cells are characterized by advanced cellular complexity, highly developed metabolic networks, and the ability to produce and excrete a wide array of redox-active secondary metabolites. These features significantly enhance their electrochemical performance in bioelectrochemical systems (BES). The process of bioelectricity generation in fungi is governed by three fundamental and interrelated mechanisms: (1) transmembrane ion transport and the establishment of electrochemical gradients across the membrane; (2) the biosynthesis and extracellular release of redox-active compounds; and (3) extracellular electron transfer (EET), which may occur through both direct and mediator-assisted pathways (Thapa et al., 2022) (**Figure 2**).

### 3.1 Ion fluxes and membrane potentials

The establishment of transmembrane electrical potential (ΔΨ) is a critical physiological characteristic of all living organisms, including fungi, serving as a foundation for intracellular communication, solute transport, and, in specific scenarios, bioelectricity production. Fungal cells regulate their membrane potential through the orchestrated movement of ions, primarily potassium (K^+^), calcium (Ca²^+^), sodium (Na^+^), and protons (H^+^), across the plasma membrane via ATP-driven pumps, voltage-gated ion channels, and exchange transporters (Kulbacka et al., 2017). In-depth investigations on *Neurospora crassa*, a model filamentous fungus, have revealed spatially restricted and temporally rhythmic Ca2^+^ fluxes at hyphal apices, which correspond with active zones of cell elongation and morphogenesis (Aramayo et al., 2013). These localized ionic pulses not only modulate polarized growth but also generate distinct electrical potential differences that can be exploited in bioelectrochemical applications. Lew (2011) further observed that *N. crassa* displays transient depolarizations at the hyphal tip (Lew, 2011), contributing to dynamic bioelectric fields essential for cellular navigation and environmental responsiveness. Electrophysiological activity has also been recorded at the macroscopic scale in higher fungi. For instance, Adamatzky (2018) identified spontaneous voltage spikes in the fruiting bodies of *Ganoderma resinaceum*, characterized by slow, action potential-like oscillations of several millivolts lasting for minutes (Adamatzky, 2018). These spikes are hypothesized to result from controlled ion fluxes across the vacuolar and plasma membranes, suggesting a sophisticated mechanism of internal electrochemical regulation. In microbial fuel cell (MFC) platforms, such ion fluxes and the resultant electrochemical gradients contribute to redox homeostasis and can enhance charge separation at the biofilm-electrode interface. Systems employing *N. crassa* biofilms have reported power densities reaching up to 280 mW/m2, with the orientation and structure of the fungal hyphae facilitating a more efficient ion-conductive interface with the anode (Luke and Burton, 2001).

### 3.2 Secretion of redox-active metabolites

A hallmark of fungal electrogenicity lies in their intrinsic capacity to biosynthesize and excrete a diverse array of redox-active metabolites such as flavins, quinones, phenolics, and organic acids that function as endogenous electron shuttles (Kracke et al., 2015; Wilhelmsen et al., 2023). These metabolites can diffuse into the MFC milieu, mediating the transfer of electrons from intracellular metabolic processes to the anode, thereby enhancing electrical output in the absence of synthetic mediators. In yeast-based MFC configurations, *Saccharomyces cerevisiae* has been documented to release extracellular NAD(P)H and other soluble cofactors that can directly reduce terminal electron acceptors, including conductive electrodes (Ostergaard et al., 2000; Sarma et al., 2021) demonstrated that *S. cerevisiae* can operate efficiently in a mediator-free MFC environment, producing power outputs of approximately 450 mW/m^2^ due to the secretion of reducing equivalents. Their findings also indicated that fermentative metabolic optimization for increased NADH production significantly boosted coulombic efficiency. Similarly, *Candida melibiosica* has been shown to release extracellular cytochrome-associated proteins and phenolic compounds that facilitate electron transfer pathways (Zhao et al., 2021). These metabolites contribute to long-term voltage stability during MFC operation, with minimal fluctuations in pH or degradation of redox-active compounds. In filamentous fungi such as *Aspergillus niger* and *Trametes versicolor*, extracellular oxidative enzymes, especially laccases and lignin peroxidases play pivotal roles as redox mediators (Andlar et al., 2018). These enzymes catalyze the oxidation of phenolic substrates while concomitantly reducing molecular oxygen, thereby serving as biocatalytic conduits for transferring electrons from fungal metabolism to the electrode. MFCs utilizing *T. versicolor* have exhibited enhanced power outputs in the range of 320–350 mW/m^2^, particularly when lignocellulosic materials such as lignin or cellulose were used as substrates (Ottoni et al., 2023). This highlights the integral role of enzyme-mediated redox cycling in augmenting bioelectricity generation. Together, the capacity of fungi to manage ion fluxes and secrete redox-active compounds positions them as versatile and efficient biocatalysts in microbial fuel cell systems.

### 3.3 Extracellular electron transfer

While the molecular underpinnings of extracellular electron transfer (EET) in fungi remain less defined compared to extensively studied bacterial systems such as *Geobacter sulfurreducens*, emerging research highlights the fungal capability to perform both direct electron transfer (DET) and mediated electron transfer (MET) to extracellular electron acceptors (Zhao et al., 2021). In DET, fungal cells engage directly with conductive surfaces, typically electrodes, through structures such as filamentous hyphae or electroactive components within the extracellular matrix, including redox-active proteins and metabolites that facilitate electron transfer (Sarma et al., 2021; Thapa et al., 2022). Evidence suggests that the complex architecture of the fungal cell wall, rich in chitin and melanin, may inherently support redox activity. Melanin pigments, which exhibit semiconductive behavior, have been detected in species like *Cryptococcus neoformans* and *Aspergillus fumigatus*, where they are implicated in oxidative stress tolerance and are also believed to mediate electron exchange processes (Lee et al., 2019a). Direct electrochemical characterization of biofilms formed by *Schizophyllum commune* and *Ganoderma lucidum* on carbon-based anodes has revealed behaviors consistent with DET (Yuan et al., 2011). Chronoamperometry and cyclic voltammetry data from these studies demonstrated current responses indicative of a direct electrical interface between the fungal biofilm and electrode, even in the absence of externally added mediators. In contrast, MET in fungi relies on the secretion of low-molecular-weight redox-active compounds that act as diffusible electron shuttles. This mechanism is particularly pronounced in white-rot fungi, such as *Phanerochaete chrysosporium*, which secrete extracellular oxidative enzymes, e.g., lignin peroxidases and manganese peroxidases, that oxidize organic substrates and facilitate electron transfer to adjacent conductive surfaces (Kersten and Cullen, 2007). Kato (2016) underscored that fungal MET processes may extend beyond energy generation to broader ecological functions, including biogeochemical redox cycling of metals such as iron and manganese in natural environments. Further support for fungal MET has been demonstrated in *Trametes pubescens*, a basidiomycete known for its lignin-degrading enzymatic arsenal. This species was observed to maintain stable current outputs over extended MFC operation periods. The sustained electrogenic activity was attributed to the secretion of redox-active enzymes, particularly laccase and veratryl alcohol oxidase, which not only mediate electron shuttling but also contribute to continuous redox cycling of extracellular metabolites as demonstrated in *Trametes pubescens* (Reyes et al., 2024).

## 4 Design and optimization of fungal-based microbial fuel cells

**Figure 3:**
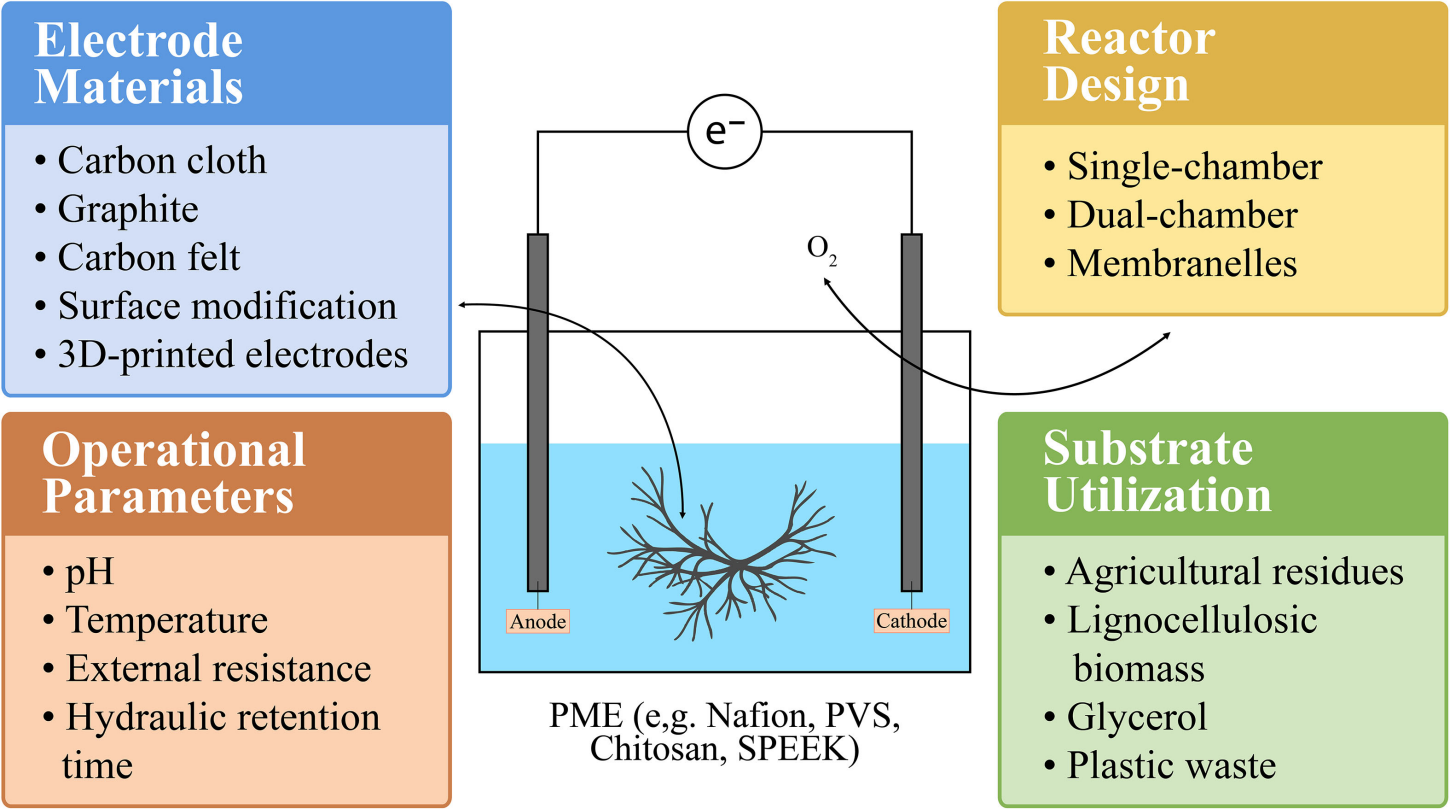
Schematic representation of design and optimization strategies in fungal-based microbial fuel cells (FMFCs). [Key components influencing performance include: (1) Electrode materials such as carbon cloth, graphite, carbon felt, surface-modified electrodes, and novel 3D-printed electrodes for enhanced surface area and biofilm attachment; (2) Proton exchange membranes (PEMs) including Nafion and alternative low-cost or biodegradable PEMs (e.g., chitosan-based, PVA-based, SPEEK); (3) Reactor design (single-chamber, dual-chamber, membraneless configurations); (4) Operational parameters (pH, temperature, external resistance, hydraulic retention time); and (5) Substrate utilization such as agricultural residues, lignocellulosic biomass, glycerol, and plastic-derived substrates].

FMFCs are an emerging category of bioelectrochemical devices that capitalize on the unique metabolic and redox characteristics of fungi for sustainable electricity production. Due to the filamentous growth patterns of fungi, their broad substrate utilization capabilities, and distinct extracellular electron transfer (EET) mechanisms, FMFC systems require specialized design and optimization strategies (**Figure 3**). Achieving optimal FMFC performance depends on fine-tuning multiple system components, including electrode composition, reactor configuration, substrate type, and operational conditions, all of which influence electron flow and overall energy yield. One of the most critical elements in FMFC optimization is the choice of electrode material. Carbon-based materials such as graphite, carbon felt, and carbon cloth are frequently employed because of their high conductivity, compatibility with biological systems, and extensive surface area, which collectively support fungal colonization and biofilm development (Mustakeem, 2015). Enhancing the electrode surface through modification with substances like poly-L-lysine, polyaniline, or metal-based nanoparticles (e.g., silver, gold, or iron oxide) has been shown to significantly improve fungal adhesion and biofilm robustness, thereby increasing the electrochemical efficiency of FMFCs (Choi et al., 2015). Additionally, integrating nanomaterials such as graphene oxide or carbon nanotubes further improves electrode conductivity and microstructural properties, facilitating enhanced electron transport from fungal cells to the electrode interface. Reactor architecture also plays a vital role in FMFC functionality. Various reactor configurations have been tested, including single-chamber, dual-chamber, and systems without membranes (Vishwanathan, 2021). Single-chamber reactors with air-cathode arrangements are particularly appealing due to their structural simplicity and reduced internal resistance, often resulting in improved power output. However, the ingress of oxygen into the reaction chamber can negatively impact the metabolism of certain fungal strains, necessitating either the selection of oxygen-tolerant species or design modifications to limit oxygen exposure (Haupt et al., 2022). Dual-chamber systems, typically separated by a proton exchange membrane (PEM), provide strictly anaerobic conditions favorable for fungal activity but may face operational limitations such as membrane clogging and increased complexity (Pal and Kumar, 2017). To overcome these challenges, alternative designs like salt bridge-mediated or membraneless FMFCs, relying on physical phase separation, have been introduced, offering more streamlined and cost-effective configurations (Umar et al., 2023). Fungal metabolic flexibility concerning carbon sources is another major advantage for FMFC deployment. Fungi are adept at decomposing a wide array of complex organic materials, including lignocellulosic biomass, agricultural by-products, and synthetic waste such as plastics. Therefore, substrate selection and pretreatment are essential components of FMFC development (Liu et al., 2014). Methods like enzymatic hydrolysis or chemical digestion can increase substrate availability, improving electron yield and power generation. Moreover, using a combination of easily degradable carbon sources (e.g., glucose) alongside more recalcitrant substrates has been shown to enhance fungal growth and stabilize current output (Wang et al., 2019). This dual-feeding strategy enables fungi to metabolize simpler substrates quickly while gradually breaking down more complex compounds, improving long-term system performance.

The performance of FMFCs is strongly influenced by operational parameters such as pH, temperature, external resistance, and hydraulic retention time (HRT). Fungal metabolism is generally most active in mildly acidic environments, with the optimal pH range for many species spanning from 4.5 to 6.5 (Ali et al., 2017). Temperature also plays a crucial role, with most fungal species exhibiting maximal growth and metabolic function between 25°C and 35°C (Dix and Webster, 1995). However, certain thermotolerant fungi are capable of sustaining activity at elevated temperatures, thereby extending the potential operating window of FMFC systems. External resistance, which governs the electrical load across the circuit, critically affects electron transfer rates. Adaptive tuning of external resistance has been reported to significantly improve power output under fluctuating environmental conditions (Sayed and Abdelkareem, 2017). In addition, adjusting the HRT influences both the duration of substrate exposure and the development of fungal biofilms on the anode surface. Longer retention times typically promote more thorough substrate degradation and enhance biofilm formation, thereby improving electron transfer efficiency and stabilizing system output. FMFCs are commonly characterized using key electrochemical metrics such as open circuit voltage (OCV), current density, and power density, typically reported in mA/m^2^ and mW/m^2^, respectively (Idris et al., 2022). Other important parameters include coulombic efficiency, which measures the proportion of electrons recovered relative to those theoretically available, and internal resistance, which provides insight into energy losses within the system (Logan, 2008). Although recent developments have reported peak power densities approaching 200 mW/m^2^ using fungi such as *Trametes versicolor*, *Penicillium chrysogenum*, and *Aspergillus niger*, these outputs remain lower than those attained with bacterial MFCs (Liu et al., 2014). This disparity underscores the need for continued innovation in enhancing fungal biofilm conductivity, optimizing electrode-microbe interactions, and refining system-level integration. To address these limitations, hybrid FMFC systems have been explored. Co-culturing fungi with other microorganisms, such as bacteria or algae, has shown synergistic benefits (Kapoore et al., 2022). Fungal-bacterial consortia leverage the complementary metabolic functions of each group, thereby enhancing substrate degradation and electron transfer. Similarly, fungal-algal systems offer added advantages by utilizing phototrophic energy from algae to sustain long-term electricity production, particularly under illuminated conditions. Moreover, advancements in modular FMFC architecture, drawing inspiration from printed circuit board designs, have enabled the development of miniaturized, stackable units suitable for decentralized energy applications. These configurations are particularly attractive for powering low-energy devices in remote or ecologically sensitive locations where conventional power infrastructure is lacking.

## 5 Integration of fungi with small-scale robotic systems

**Figure 4:**
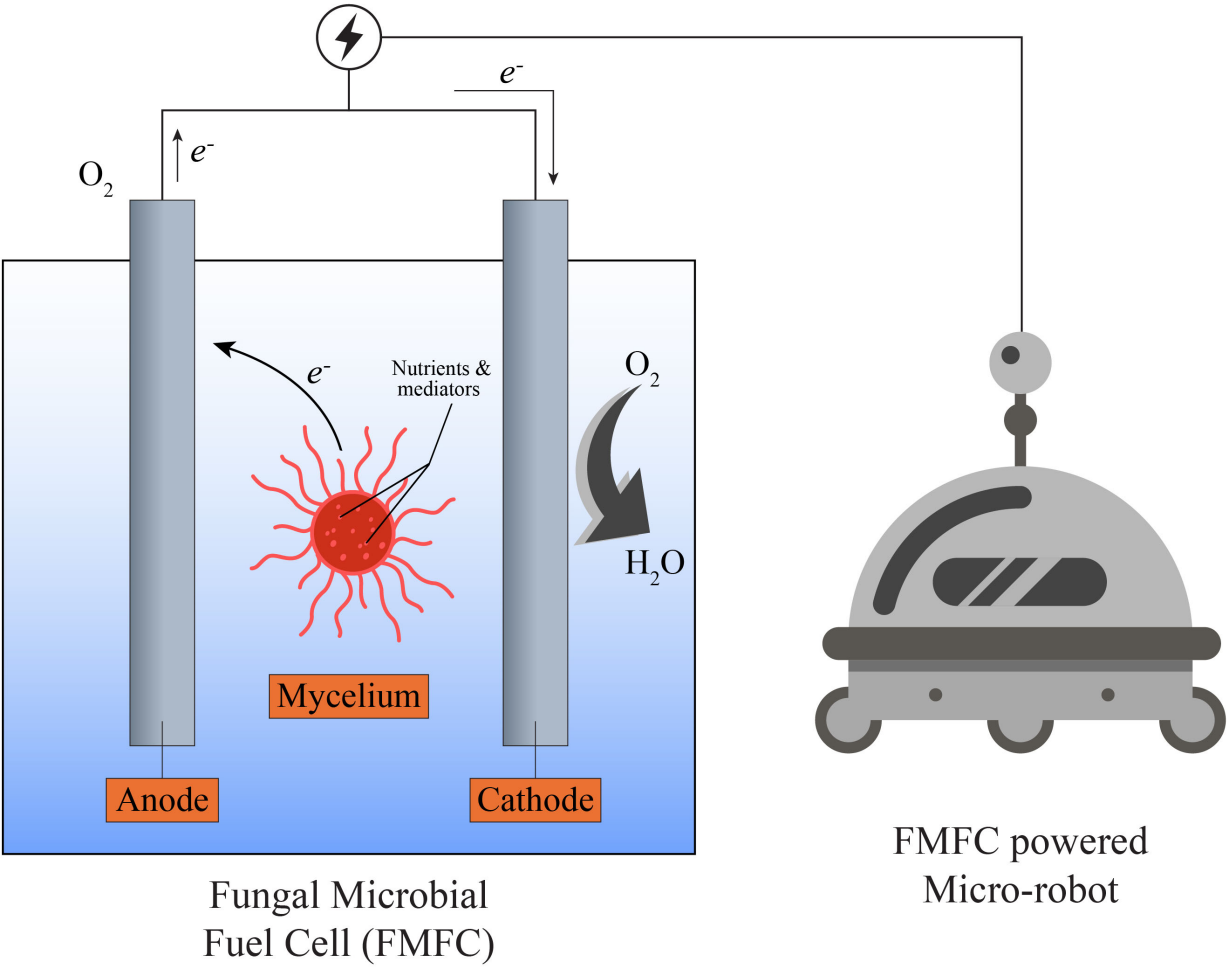
Schematic representation of a fungal microbial fuel cell (FMFC) integrated as an onboard power module within a micro-robot [The fungal FMFC contains a mycelium-based anode chamber and a corresponding cathode chamber, where fungal metabolic activity generates electron flow to the external circuit. Electrical output is routed through conductive wiring to the micro-robot’s internal power system. Arrows indicate the direction of electron transfer. The illustration is conceptual and demonstrates the placement and functional linkage of the fungal MFC within the robot chassis].

Fungi have traditionally been recognized for their significant contributions to bioenergy and environmental sustainability, owing to their exceptional biochemical capabilities in decomposing organic matter and transforming waste into valuable by-products. Species such as *Pleurotus ostreatus* and *Ganoderma lucidum* have been extensively investigated for their efficiency in converting lignocellulosic biomass into biofuels, including bioethanol and biogas (Cherukuri and Akkina, 2019). Notably, *Pleurotus ostreatus* has demonstrated a conversion efficiency of approximately 50–60% when processing agricultural waste materials into usable biofuels (Girmay et al., 2016). This bioconversion is primarily facilitated by the fungal mycelium, a vegetative network of hyphal threads that enzymatically deconstructs complex polymers into simpler, metabolizable compounds, rendering it integral to sustainable bioenergy systems. Beyond conventional bioenergy applications, recent advances in robotics and bioengineering have catalyzed a new wave of research focused on the integration of fungal systems, particularly their mycelial networks, into robotic technologies. This emerging interdisciplinary field explores the use of fungi as biological substrates in the development of adaptive, environmentally responsive, and sustainable robotic platforms (**Figure 4**). The mycelial network, owing to its intrinsic capabilities for environmental sensing, self-organization, and structural plasticity, provides a biologically inspired framework for the design of soft robotics and biohybrid systems. A compelling example of this innovation is the development of biohybrid robots, which integrate living fungal tissues or mycelial threads with synthetic robotic components (Lin et al., 2022; Gantenbein et al., 2023). These biohybrid constructs bridge biological and artificial systems, enabling robotic platforms that can perceive and respond to environmental cues with enhanced autonomy and adaptability. Traditional robotic systems, which often rely on rigid metals and synthetic polymers, face limitations in environments requiring resilience, repairability, and responsive behavior (Tejada et al., 2025). In contrast, mycelium offers a flexible, self-healing architecture capable of sensing multiple stimuli simultaneously, thereby surpassing the limitations of conventional electromechanical sensors. Fungi-based robotic systems exploit the natural electrical activity of mycelium to achieve motion control and environmental interaction (Mishra et al., 2024). Mycelia, which structurally and functionally resemble neural networks, can propagate electrical impulses in response to external stimuli. This electrophysiological property forms the basis for fungal control over robotic actuators and motion systems. In recent experimental setups, researchers have demonstrated that targeted ultraviolet (UV) light exposure can modulate the activity of mycelium-infused robots, altering their mechanical behaviors in real-time (Degeurin, 2024). For instance, UV exposure was shown to modify crawling patterns in soft-bodied robots and adjust rolling speed and directional control in wheeled robotic units (Mishra et al., 2024). These dynamic responses occur because the electrical signals generated by the mycelium directly influence the motors and actuators responsible for robotic locomotion. Successful signal transduction in such systems depends critically on the strategic placement of electrodes, electrically conductive interfaces that must be delicately affixed to specific regions of the mycelial network. These regions are often exceedingly thin and fragile, requiring precision in electrode integration to ensure reliable data acquisition. Once connected, these electrodes detect the electrophysiological activity of the mycelium, which is subsequently converted into digital input, enabling the robotic system to process and react to environmental stimuli.

**Figure 5:**
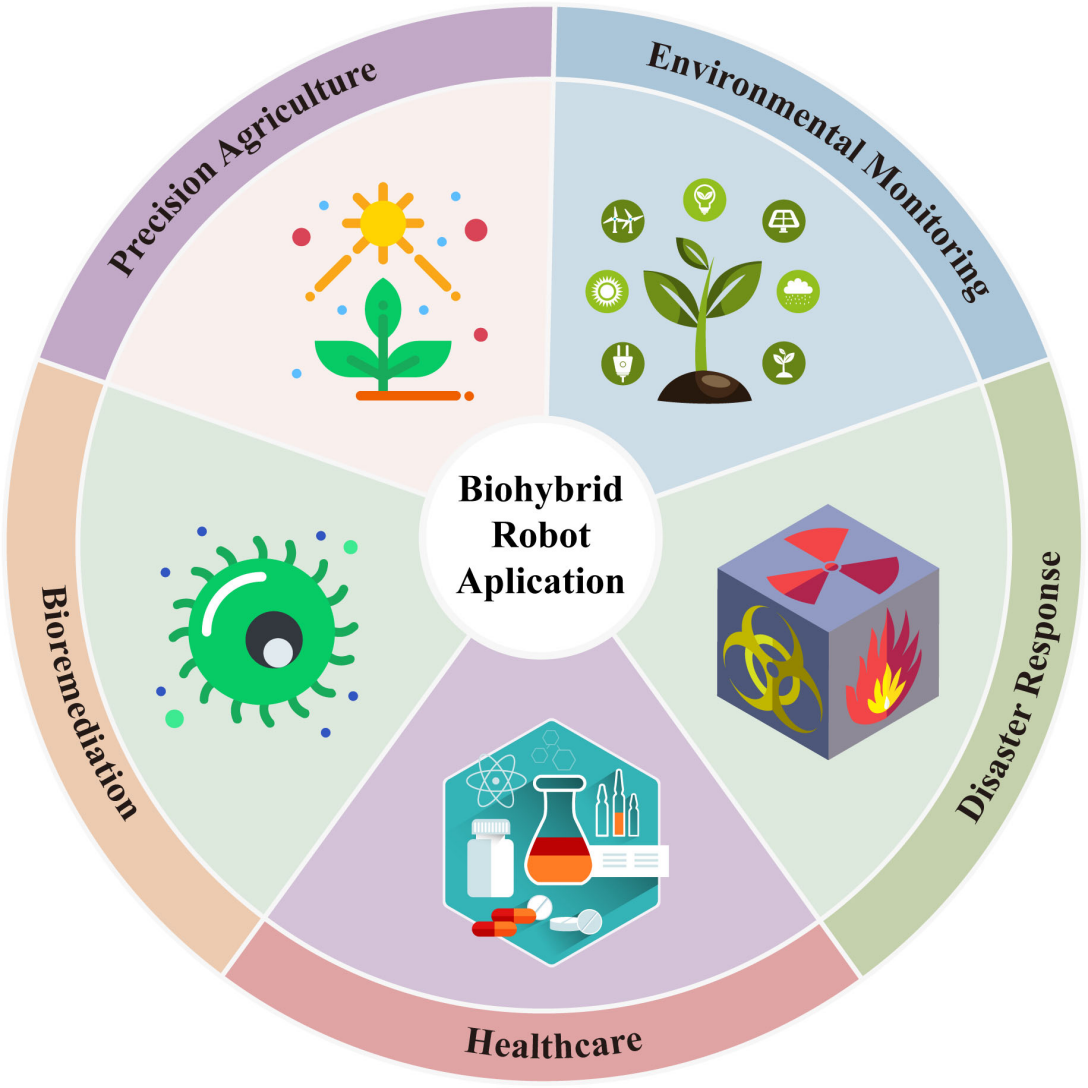
Overview of major application domains of biohybrid robots.

The most recent and remarkable advancement in this emerging field originates from an unconventional biological source: the King Oyster Mushroom. Researchers at Cornell University have successfully developed biohybrid robots, termed “shroom bots”, by integrating the mycelium of *Pleurotus eryngii* into robotic systems. Crucially, it is not the fruiting body but the mycelial network that is utilized, as it generates bioelectrical signals during growth. These signals are harnessed through embedded electrodes and converted into movement commands, enabling the robot to respond to environmental stimuli such as ultraviolet light. Acting simultaneously as both a sensory and processing unit, the mycelium empowers these robots with an intrinsic ability to perceive and interact with their surroundings in a manner fundamentally distinct from conventional robotics (Jithin, 2024). This biohybrid paradigm signifies a significant leap in robotic adaptability. Soft actuators composed of mycelium have been engineered to respond to variations in temperature, humidity, and light, thereby allowing robotic systems to function more autonomously in dynamic settings. Mishra et al. (2024) notably constructed two such robots: a spider-like soft robot and a wheeled hard robot, both driven by the electrophysiological signals of living mycelia (Mishra et al., 2024). These signals, akin to action potentials, regulate the robots’ motors and valves. Upon exposure to UV light, the robots demonstrated not only locomotion but also modifications in gait and rolling dynamics, highlighting the mycelium’s capacity for nuanced environmental responsiveness. The potential of mycelium as a biological actuator is further supported by the findings of Vašatko et al. (2022), who demonstrated that mycelium structures could achieve up to a 40% change in length under alternating wet and dry conditions (Vašatko et al., 2022). This hygroscopic response is particularly valuable for applications in agriculture, soil health monitoring, and environmental remediation, where adaptability to changing moisture levels is crucial. Mycelium-based robots could be engineered to operate in polluted or degraded environments, autonomously adjusting their behavior in response to the presence of contaminants or specific environmental cues. Expanding on this potential, researchers at Harvard University’s Fungal Robotics initiative unveiled a groundbreaking soft autonomous robot in 2020, constructed entirely from living *Ganoderma lucidum* mycelium seeded onto a 3D-printed scaffold. The robot’s motion was actuated by the growth-induced deformation of the scaffold in response to humidity. This approach demonstrated not only movement and object manipulation but also the capacity for function within fragile ecosystems, an innovation at the intersection of biohybrid mechanics and ecological sensitivity (Lin et al., 2022).

Beyond their structural and mechanical utility, fungi are renowned for their bioremediation capabilities. Mycelium’s capacity to degrade environmental pollutants is particularly promising. *Pleurotus ostreatus*, for instance, has been documented to reduce petroleum-derived pollutant concentrations by up to 90% within 30 days in contaminated environments (Ekundayo, 2014). This capability could be embedded within robotic systems to create mobile units capable of oil spill cleanup, plastic degradation, or filtration of heavy metals and toxins from soil and water. Such systems promise a novel convergence of environmental robotics and biological sustainability. Agriculture stands to benefit considerably from mycelium-integrated robotics. In a study by Rus and Tolley (Rus and Tolley, 2015), soft robotic elements composed of mycelium-based materials were demonstrated to interact with delicate crops without inflicting damage. These biohybrid tools could be applied in precision agriculture to monitor plant health, facilitate pollination, or detect early signs of pest infestation and disease. Their soft, pliable structures provide a clear advantage over rigid traditional robots, particularly in the manipulation of sensitive plant tissues. Furthermore, the regenerative ability of mycelium introduces a unique self-healing property, enhancing robot durability while minimizing operational costs and ecological waste.

## 6 Applications of biohybrid robots

The development of biohybrid robots offers unique capabilities to address contemporary challenges across various industries. These robots leverage the natural properties of fungal mycelium, such as self-healing, environmental adaptability, and responsiveness to various stimuli, including light, humidity, and chemical fluctuations. These biological characteristics enable biohybrid systems to offer sustainable, efficient, and adaptive solutions to problems in precision agriculture, environmental monitoring, disaster recovery, space exploration, and other domains (**Figure 5**). In precision agriculture, biohybrid robots present a transformative approach to crop management (Taha et al., 2025). Traditional farming practices often rely on manual observation and broad-spectrum chemical tests to assess soil health, which can be inefficient and environmentally intrusive. Biohybrid robots incorporating fungal mycelium offer enhanced sensing capabilities because the mycelial network generates bioelectrical responses to subtle changes in moisture, pH, nutrient availability, and chemical stressors (Mazzolai and Laschi, 2020). Current evidence suggests that mycelium-based sensors can detect low-intensity environmental fluctuations with higher sensitivity than conventional robotic sensors, particularly in detecting diffuse chemical gradients and micro-scale soil heterogeneity (Mishra et al., 2024). Phillips et al. (2023) demonstrated that mycelium-bound composites generate distinct electrical spikes in response to small moisture fluctuations. Spontaneous spike trains appeared when moisture content shifted between 95–65% in fresh composites and 15–5% in partially dried ones- ranges where conventional soil sensors show limited responsiveness. Even single water droplets deposited on the surface triggered measurable electrical activity, highlighting micro-scale moisture-detection abilities far more sensitive than typical electronic moisture probes. While traditional robotic sensing systems remain superior for high-resolution quantitative measurements (Jain, 2025), biohybrid robots excel in continuous, distributed, and passive environmental sensing due to the natural signal-processing capacity of fungal networks (Webster-Wood et al., 2022). This enhanced sensing precision could optimize agricultural inputs and reduce chemical use, helping to minimize nutrient runoff and secondary environmental impacts such as algal blooms. Studies have demonstrated the viability of using mycelium in robotic systems, with *Pleurotus eryngii* (King Oyster Mushroom) being integrated into biohybrid systems to create adaptive sensors that guide robotic movement and environmental interactions (Jithin, 2024). The environmental monitoring applications of biohybrid robots also hold great promise, particularly in the context of real-time ecological assessments. These robots, using mycelium-based sensors, can be deployed across a wide range of ecosystems to monitor pollutants, changes in soil health, and shifts in environmental conditions. For instance, aquatic biohybrid robots could be used to monitor coral reef health and assess water quality by sensing pollutants or changes in pH levels (Piñeros et al., 2024). On land, these robots could detect air pollutants and track soil degradation, providing valuable data for ecosystem management and conservation. Their biological adaptability makes them highly versatile, capable of functioning in diverse environments and supporting ecological monitoring efforts. In disaster response, biohybrid robots offer a new frontier for search and rescue operations. Unlike traditional robots that rely solely on synthetic sensors, mycelium-integrated robots can also detect diffuse biological or chemical cues, such as metabolic volatiles or moisture signatures associated with human presence. However, synthetic sensors remain superior in precision, speed, and quantitative accuracy, especially for parameters such as gas concentration, temperature, and structural integrity (Chen et al., 2024). Biohybrid sensors, by contrast, excel in detecting low-intensity, spatially distributed, or organic chemical gradients, offering complementary capabilities rather than replacing traditional systems (Oda et al., 2021). For example, following a seismic event, these robots could navigate through rubble, using their mycelium networks to detect faint chemical markers or biological indicators that signify the presence of trapped individuals (Oberdick, 2025). This ability would allow these robots to access environments that are otherwise too hazardous or challenging for human rescuers, providing a more effective response to natural disasters. The mycelium’s sensitivity to environmental changes and its ability to adapt to varying conditions make these biohybrids especially suitable for this application (Mishra et al., 2024).

Space exploration is another area where biohybrid robots, powered by mycelium, could revolutionize current practices (Lipińska et al., 2022; Montana-Hoyos et al., 2022). Mycelium’s ability to withstand extreme conditions, such as high radiation levels and extreme temperatures, makes it a prime candidate for extraterrestrial missions. Biohybrid robots could be deployed in harsh environments like the surface of Mars. These robots would not only assist in exploring these remote regions but could also be utilized for habitat construction or *in-situ* resource utilization, using mycelium-based materials to build and repair structures on extraterrestrial surfaces. The ability of mycelium to grow and adapt in resource-scarce environments would provide a sustainable and self-repairing solution for space missions, significantly contributing to future space exploration. Beyond environmental and disaster response, biohybrid robots also offer promising applications in healthcare and construction (Zarepour et al., 2024). In healthcare, these robots could be developed for human tissue interaction, including applications in surgery, prosthetics, and drug delivery. The inherent self-healing properties of mycelium could reduce the need for replacements in medical devices, thus providing longer-lasting, cost-effective solutions. In the construction industry, biohybrid robots could be used to create self-repairing, adaptive structures. Mycelium’s ability to create biodegradable and fire-resistant materials is being explored for use in building insulation, offering sustainable alternatives to traditional materials. Additionally, the use of mycelium in construction could lead to the development of more resilient buildings capable of responding to environmental changes, further advancing the field of sustainable architecture (Derme et al., 2024). One of the most exciting and environmentally impactful applications of biohybrid robots lies in bioremediation (Webster-Wood et al., 2022). Mycelium has long been recognized for its ability to degrade organic pollutants, including hydrocarbons and heavy metals, through its natural enzymatic activity. This makes it an ideal candidate for use in robots designed for environmental cleanup. Mycelium-based robots could be deployed to clean oil spills, degrade plastic waste, or filter toxic chemicals from contaminated water sources. These robots would provide a sustainable and biologically driven solution to some of the most pressing environmental challenges. Research has shown that *Pleurotus ostreatus*, a type of fungus, can degrade petroleum-based pollutants by up to 90% in just a month, demonstrating the potential for mycelium in large-scale environmental remediation.

## 7 Challenges and future perspectives of biohybrid fungal mycelium robotics

The integration of fungal mycelium into robotics presents exciting prospects but also raises a host of ethical and practical challenges. As with any emerging technology, it is essential to balance innovation with responsible use, considering both the potential benefits and the broader ecological, ethical, and practical implications. One of the primary challenges in the development of biohybrid robots is the lack of standardized design and modeling tools. Existing frameworks must address not only the actuation effects of mycelium on robotic movement but also the complex interactions within microorganism swarms i.e., large coordinated microbial communities that exhibit collective behaviours such as synchronized growth, chemical signalling, and distributed sensing that can influence or enhance biohybrid system performance. Data-driven approaches hold promise for modeling these systems, yet they often rely on extensive datasets, which can be difficult to generate and standardize effectively (Leaman et al., 2020). The ecological risks of deploying mycelium-powered robots depend strongly on the fungal species used. Potential impacts vary with whether the fungus is pathogenic or non-pathogenic and whether it can survive or reproduce outside the robots’ controlled environment, given its limits for temperature, pH, and moisture. These risks can be substantially reduced by selecting non-pathogenic strains with narrow environmental tolerances that are unlikely to persist independently, while still providing adequate bioelectrical performance. Such selective strain choice offers a practical approach to minimizing ecological disruption during field deployment. Furthermore, the introduction of artificial biological entities into the trophic chain could blur the lines between living and non-living components of ecosystems, potentially causing long-term disruptions. Researchers must thoroughly evaluate the ecological impact of these technologies to ensure that their deployment does not harm natural habitats. Ethical concerns surrounding the use of fungal mycelium in robotics must be placed in context. Humans have harnessed microorganisms, including fungi, for millennia in fermentation, food production, and medicine, and their use in technology is not inherently morally problematic. The ethical focus therefore does not stem from using fungi per se, but from ensuring that biohybrid robotic systems do not introduce pathogenic strains, environmentally persistent organisms, or genetically engineered vectors into natural ecosystems. Accordingly, the need for guidelines arises from biosafety, ecological stewardship, and responsible deployment, rather than from moral objections to using fungal life in technological applications. Continuous dialogue among policymakers, biosafety experts, ethicists, and researchers remains essential to establish robust standards that safeguard environmental and public health while enabling responsible innovation.

Looking to the future, the potential for biohybrid robotics remains promising. With continued research and development, these technologies will likely evolve to become more autonomous, capable of operating independently in diverse environments. Advances in interfacing technologies will enhance the responsiveness and efficiency of biohybrids, allowing them to perform more intricate and precise tasks. A particularly exciting direction is the integration of artificial intelligence (AI) and machine learning with biohybrid systems. AI algorithms could be used to process the electrical signals produced by mycelium, enabling robots to adapt their behaviors over time and perform complex, multi-faceted tasks. This fusion of biological and artificial intelligence could lead to robots that are not only more adaptable but also capable of addressing a wide range of challenges across various sectors. As technology matures, applications for biohybrid robots will expand significantly. In agriculture, these robots could monitor soil health, plant seeds, and assist in harvesting, while in environmental monitoring they could form networks that track climate, pollution, and ecosystem changes. In disaster response, swarms of biohybrids could use their natural sensitivity to locate survivors. Unlike existing robots, biohybrid systems offer advantages derived from their living components such as adaptive growth, self-healing, ultra-low energy needs, and stable sensing in harsh or dynamic environments, making them a complementary tool rather than a replacement for traditional robotic platforms. Henceforth, the integration of mycelium into robotic systems represents a promising avenue for advancing both technology and sustainability, with the potential to address some of the most pressing challenges faced by modern society.

## 8 Conclusion

Fungal bioelectricity presents a promising and sustainable approach to powering small-scale robotic systems, utilizing the unique conductive properties of fungal mycelium in microbial fuel cells (MFCs). Although fungal-based microbial fuel cells (FMFCs) demonstrate significant potential, challenges surrounding energy efficiency, scalability, and system integration remain. To enhance their effectiveness, future studies should focus on optimizing electrode materials, improving microbial interactions, and refining FMFC design. Moreover, exploring the integration of fungi with robotic systems could open up new opportunities for autonomous robots, environmental monitoring, and self-sustaining technologies. Progress in these areas will pave the way for establishing fungal bioelectricity as a reliable, eco-friendly energy source for biohybrid robots, contributing to the advancement of renewable energy solutions and sustainable robotic systems.

## References

[B1] Adamatzky A. (2018). On spiking behaviour of oyster fungi *Pleurotus djamor*. Sci. Rep. 8, 1–7. doi: 10.1038/s41598-018-26007-1, PMID: 29777193 PMC5959856

[B2] Afroz A. S. Romano D. Inglese F. Stefanini C. (2021). Towards bio-hybrid energy harvesting in the real world: Pushing the boundaries of technologies and strategies using bio-electrochemical and bio-mechanical processes. Appl. Sci. 11, 2220. doi: 10.3390/app11052220

[B3] Ali J. Sohail A. Wang L. Haider M. R. Mulk S. Pan G. (2018). Electro-microbiology as a promising approach towards renewable energy and environmental sustainability. Energies 11, 1822. doi: 10.3390/en11071822

[B4] Ali M. R. S. Sadiq F. J. Ahmed F. Al-Aaraji A. M. (2017). *Effect of some physical factors on growth of five fungal* sp*ecies.* ( ResearchGate). Available online at: https://www.researchgate.net/publication/332222734 (Accessed September 24, 2025).

[B5] Allen R. M. Bennetto H. P. (1993). Microbial fuel cells: Electricity production from carbohydrates. Appl. Biochem. Biotechnol. 39–40, 27–40. doi: 10.1007/BF02918975

[B6] Andlar M. Rezić T. Marđetko N. Kracher D. Ludwig R. Šantek B. (2018). Lignocellulose degradation: An overview of fungi and fungal enzymes involved in lignocellulose degradation. Eng. Life Sci. 18, 768–778. doi: 10.1002/elsc.201800039, PMID: 32624871 PMC6999254

[B7] Apollon W. (2023). An overview of microbial fuel cell technology for sustainable electricity production. Membranes 13, 884. doi: 10.3390/membranes13110884, PMID: 37999370 PMC10672772

[B8] Apollon W. Valera-Montero L. L. Perales-Segovia C. Maldonado-Ruelas V. A. Ortiz-Medina R. A. Gómez-Leyva J. F. . (2022). Effect of ammonium nitrate on novel cactus pear genotypes aided by biobattery in a semi-arid ecosystem. Sustain. Energy Technol. Assessments 49, 101730. doi: 10.1016/j.seta.2021.101730

[B9] Aramayo R. Selker E. U. Allis D. Caparros M.-L. Jenuwein T. Reinberg D. (2013). *Neurospora crassa*, a model system for epigenetics research. Cold Spring Harbor Perspect. Biol. 5, a017921. doi: 10.1101/cshperspect.a017921, PMID: 24086046 PMC3783048

[B10] Balagurunathan R. Radhakrishnan M. Shanmugasundaram T. Gopikrishnan V. Jerrine J. (2020). “ Evaluation of actinobacteria for bioenergy applications,” in Protocols in Actinobacterial Research. (Cham, Switzerland: Springer Nature) 205–210. doi: 10.1007/978-1-0716-0728-2_14

[B11] Beliaev A. S. Nealson K. H. Pinchuk G. Rodrigues J. L. M. Saffarini D. Serres M. H. . (2008). “ Towards environmental systems biology of *Shewanella.*,” in Nature Reviews Microbiology. (London, United Kingdom: Nature Publishing Group). Available online at: https://www.osti.gov/biblio/1045271 (Accessed September 23, 2025)., PMID: 10.1038/nrmicro1947 18604222

[B12] Bond D. R. Lovley D. R. (2003). Electricity production by *Geobacter sulfurreducens* attached to electrodes. Appl. Environ. Microbiol. 69, 1548–1555. doi: 10.1128/AEM.69.3.1548-1555.2003, PMID: 12620842 PMC150094

[B13] Chaijak P. Sukkasem C. Lertworapreecha M. Boonsawang P. Wijasika S. Sato C. (2018). Enhancing electricity generation using a laccase-based microbial fuel cell with yeast *Galactomyces reessii* on the cathode. J. Microbiol. Biotechnol. 28, 1360–1366. doi: 10.4014/JMB.1803.03015, PMID: 30021424

[B14] Chen L. Xia C. Zhao Z. Fu H. Chen Y. (2024). AI-driven sensing technology: Review. Sensors 24, 2958. doi: 10.3390/s24102958, PMID: 38793814 PMC11125233

[B15] Cherukuri P. J. Akkina R. C. (2019). “ Bioconversion of biomass to biofuel using fungal consortium,” in Recent advancement in white biotechnology through fungi. Eds. Yadav A. Singh S. Mishra S. Gupta A. ( Springer), 381–396. doi: 10.1007/978-3-030-25506-0_15

[B16] Choi Y. Yagati A. K. Cho S. (2015). Electrochemical characterization of poly-L-lysine coating on indium tin oxide electrode for enhancing cell adhesion. J. Nanoscience Nanotechnology 15, 7881–7885. doi: 10.1166/JNN.2015.11229, PMID: 26726433

[B17] Choudhary M. Verma P. Ray S. (2024). A comprehensive review on bio-electrochemical systems for wastewater treatment: Process, electricity generation and future aspect. Environment Dev. Sustainability, 1–30. doi: 10.1007/s10668-024-05866-x

[B18] Christwardana M. Frattini D. Accardo G. Yoon S. P. Kwon Y. (2018). Effects of methylene blue and methyl red mediators on performance of yeast-based microbial fuel cells adopting polyethylenimine-coated carbon felt as anode. J. Power Sources 396, 1–11. doi: 10.1016/j.jpowsour.2018.06.005

[B19] Cohen B. (1931). The bacterial culture as an electrical half-cell. Available online at: https://scholar.google.com/scholar_lookup?title=The%20bacterial%20culture%20as%20an%20electrical%20half-cell&publication_year=1931&author=B.%20Cohen (Accessed September 24, 2025).

[B20] Coursolle D. Baron D. B. Bond D. R. Gralnick J. A. (2010). The Mtr respiratory pathway is essential for reducing flavins and electrodes in *Shewanella oneidensis*. J. Bacteriology 192, 467–474. doi: 10.1128/JB.00925-09, PMID: 19897659 PMC2805334

[B21] Cui Y. Rashid N. Hu N. Rehman M. S. U. Han J. I. (2014). Electricity generation and microalgae cultivation in microbial fuel cell using microalgae-enriched anode and bio-cathode. Energy Conversion Manage. 79, 674–680. doi: 10.1016/j.enconman.2013.12.032

[B22] Da Costa C. Hadiyanto (2018). Bioelectricity production from microalgae-microbial fuel cell technology (MMFC). MATEC Web Conferences 156, 1017. doi: 10.1051/matecconf/201815601017

[B23] Davis J. B. (1963). Generation of electricity by microbial action. Adv. Appl. Microbiol. 5, 51–64. doi: 10.1016/S0065-2164(08)70006-6, PMID: 14149666

[B24] Davis J. B. Yarbrough H. F. (1962). Preliminary experiments on a microbial fuel cell. Science 137, 615–616. doi: 10.1126/science.137.3530.615, PMID: 17836545

[B25] Degeurin M. (2024). This robot is being controlled by a king oyster mushroom. Sci. Robotics 9. doi: 10.1126/scirobotics.adk8019, PMID: 39196952

[B26] Derme T. Schwarze F. W. M. R. Dillenburger B. (2024). Understanding the role of controlled environments for producing mycelium-bound composites: Advancing circular practices for integrating biotechnology into the construction industry. Global Challenges 8, 2300197. doi: 10.1002/gch2.202300197, PMID: 39006056 PMC11237183

[B27] Dix N. J. Webster J. (1995). “ Fungi of extreme environments,” in Fungal ecology. (Dordrecht, The Netherlands: Springer Science+Business Media) 322–340. doi: 10.1007/978-94-011-0693-1_12

[B28] Ekundayo F. O. (2014). Comparative studies on biodegradative abilities of *Pleurotus ostreatus* and *P. pulmonarius* in soils contaminated with crude and used engine oils. Adv. Microbiol. 4, 849–855. doi: 10.4236/aim.2014.412094

[B29] Elmekawy A. Hegab H. M. Vanbroekhoven K. Pant D. (2014). Techno-productive potential of photosynthetic microbial fuel cells through different configurations. Renewable Sustain. Energy Rev. 39, 617–627. doi: 10.1016/j.rser.2014.07.116

[B30] Enamala M. K. Enamala S. Chavali M. Donepudi J. Yadavalli R. Kolapalli B. . (2018). Production of biofuels from microalgae: A review on cultivation, harvesting, lipid extraction, and numerous applications of microalgae. Renewable Sustain. Energy Rev. 94, 49–68. doi: 10.1016/j.rser.2018.05.012

[B31] Freguia S. Masuda M. Tsujimura S. Kano K. (2009). *Lactococcus lactis* catalyses electricity generation at microbial fuel cell anodes via excretion of a soluble quinone. Bioelectrochemistry 76, 14–18. doi: 10.1016/j.bioelechem.2009.04.001, PMID: 19411192

[B32] Gantenbein S. Colucci E. Käch J. Trachsel E. Coulter F. B. Rühs P. A. . (2023). Three-dimensional printing of mycelium hydrogels into living complex materials. Nat. Materials 22, 128–134. doi: 10.1038/s41563-022-01429-5, PMID: 36550372

[B33] Gao K. Lu Y. (2021). Putative extracellular electron transfer in methanogenic archaea. Front. Microbiol. 12. doi: 10.3389/fmicb.2021.611739, PMID: 33828536 PMC8019784

[B34] Garbini G. L. Barra Caracciolo A. Grenni P. (2023). Electroactive bacteria in natural ecosystems and their applications in microbial fuel cells for bioremediation: A review. Microorganisms 11, 1255. doi: 10.3390/microorganisms11051255, PMID: 37317229 PMC10263229

[B35] Garimella S. S. S. Rachakonda S. V. Pratapa S. S. Mannem G. D. Mahidhara G. (2024). From cells to power cells: Harnessing bacterial electron transport for microbial fuel cells (MFCs). Ann. Microbiol. 74, 15. doi: 10.1186/s13213-024-01761-y

[B36] Girmay Z. Gorems W. Birhanu G. Zewdie S. (2016). Growth and yield performance of *Pleurotus ostreatus* (Jacq. Fr.) Kumm (oyster mushroom) on different substrates. AMB Express 6, 87. doi: 10.1186/s13568-016-0265-1, PMID: 27704469 PMC5050175

[B37] Gunawardena A. Fernando S. To F. (2008). Performance of a yeast-mediated biological fuel cell. Int. J. Mol. Sci. 9, 1893–1907. doi: 10.3390/ijms9101893, PMID: 19325724 PMC2635613

[B38] Hassan R. Y. A. Febbraio F. Andreescu S. (2021). Microbial electrochemical systems: Principles, construction and biosensing applications. Sensors 21, 1279. doi: 10.3390/s21041279, PMID: 33670122 PMC7916843

[B39] Haupt D. R. Landwehr L. Schumann R. Hahn L. Issa M. Coskun C. . (2022). A new reactor concept for single-chamber microbial fuel cells and possible anti-fouling strategies for long-term operation. Microorganisms 10, 2421. doi: 10.3390/microorganisms10122421, PMID: 36557674 PMC9784785

[B40] Holmes D. E. Ueki T. Tang H. Y. Zhou J. Smith J. A. Chaput G. . (2019). A membrane-bound cytochrome enables *Methanosarcina acetivorans* to conserve energy from extracellular electron transfer. mBio 10, e00789–e00719. doi: 10.1128/mBio.00789-19, PMID: 31431545 PMC6703419

[B41] Hu Y. Wang Y. Han X. Shan Y. Li F. Shi L. (2021). Biofilm biology and engineering of *Geobacter* and Shewanella spp. for energy applications. Front. Bioengineering Biotechnol. 9. doi: 10.3389/fbioe.2021.786416, PMID: 34926431 PMC8683041

[B42] Hubenova Y. Mitov M. (2015). Extracellular electron transfer in yeast-based biofuel cells: A review. Bioelectrochemistry 106, 177–185. doi: 10.1016/j.bioelechem.2015.04.001, PMID: 25887619

[B43] Idris M. O. Yaqoob A. A. Ibrahim M. N. M. Noh N. A. M. Daud N. N. M. (2022). “ Electrochemical measurements of microbial fuel cells (MFCs),” in Microbial Fuel Cell Technology for Bioelectricity ( Springer), 41–64. doi: 10.1007/978-981-19-2681-5_4

[B44] Ikeda S. Takamatsu Y. Tsuchiya M. Suga K. Tanaka Y. Kouzuma A. . (2021). *Shewanella oneidensis* MR-1 as a bacterial platform for electro-biotechnology. Essays Biochem. 65, 355–364. doi: 10.1042/EBC20200178, PMID: 33769488 PMC8314016

[B45] Jain K. S. (2025). The rise of sensors for robotics in real-world applications: A technical review. Eur. J. Comput. Sci. Inf. Technol. 13, 30–41. doi: 10.37745/ejcsit.2013/vol13n363041

[B46] Jatoi A. S. Hashmi Z. Mazari S. A. Mubarak N. M. Karri R. R. Ramesh S. . (2022). A comprehensive review of microbial desalination cells for present and future challenges. Desalination 535, 115808. doi: 10.1016/j.desal.2022.115808

[B47] Ji B. Zhao Y. Yang Y. Li Q. Man Y. Dai Y. . (2023). Curbing per- and polyfluoroalkyl substances (PFASs): First investigation in a constructed wetland–microbial fuel cell system. Water Res. 230, 119530. doi: 10.1016/j.watres.2022.119530, PMID: 36577258

[B48] Jithin (2024). Living robots: The fusion of fungus and technology in new robotic designs. ( RootsAid). Available online at: https://rootsaid.com/living-robots-fungus-and-technology/ (Accessed December 27, 2025).

[B49] Kapoore R. V. Padmaperuma G. Maneein S. Vaidyanathan S. (2022). Co-culturing microbial consortia: Approaches for applications in biomanufacturing and bioprocessing. Crit. Rev. Biotechnol. 42, 46–72. doi: 10.1080/07388551.2021.1921691, PMID: 33980092

[B50] Kato S. (2016). Microbial extracellular electron transfer and its relevance to iron corrosion. Microbial Biotechnol. 9, 141–148. doi: 10.1111/1751-7915.12340, PMID: 26863985 PMC4767289

[B51] Kersten P. Cullen D. (2007). Extracellular oxidative systems of the lignin-degrading Basidiomycete *Phanerochaete chrysosporium*. Fungal Genet. Biol. 44, 77–87. doi: 10.1016/j.fgb.2006.07.007, PMID: 16971147

[B52] Khan M. J. Das S. Vinayak V. Pant D. Ghangrekar M. M. (2022). Live diatoms as potential biocatalyst in a microbial fuel cell for harvesting continuous diafuel, carotenoids, and bioelectricity. Chemosphere 291, 132841. doi: 10.1016/j.chemosphere.2021.132841, PMID: 34767852

[B53] Kim B. Kim H. Hyun M. S. (1999). Direct electrode reaction of Fe(III)-reducing bacterium *Shewanella putrefaciens*. J. Microbiol. Biotechnol. Available online at: https://scholar.kyobobook.co.kr/article/detail/4040012723950.

[B54] Koch C. Harnisch F. (2016). Is there a specific ecological niche for electroactive microorganisms? ChemElectroChem 3, 1282–1295. doi: 10.1002/celc.201600079

[B55] Kracke F. Vassilev I. Krömer J. O. (2015). Microbial electron transport and energy conservation: The foundation for optimizing bioelectrochemical systems. Front. Microbiol. 6. doi: 10.3389/fmicb.2015.00575, PMID: 26124754 PMC4463002

[B56] Kruzic A. P. Kreissl J. F. (2009). Natural treatment and onsite systems. Water Environ. Res. 81. doi: 10.2175/106143009X12445568399659

[B57] Kulbacka J. Choromańska A. Rossowska J. Saczko J. Rols M. P. (2017). “ Cell membrane transport mechanisms: Ion channels and electrical properties of cell membranes,” in Advances in Anatomy, Embryology and Cell Biology. (Cham, Switzerland: Springer International Publishing) 227, 39–58. doi: 10.1007/978-3-319-56895-9_3, PMID: 28980039

[B58] Kumar S. S. Malyan S. K. Basu S. Bishnoi N. R. (2017). Syntrophic association and performance of *Clostridium*, *Desulfovibrio*, *Aeromonas* and *Tetrathiobacter* as anodic biocatalysts for bioelectricity generation in a dual-chamber microbial fuel cell. Environ. Sci. pollut. Res. 24, 16019–16030. doi: 10.1007/s11356-017-9112-4, PMID: 28537018

[B59] Leaman E. J. Sahari A. Traore M. A. Geuther B. Q. Morrow C. M. Behkam B. (2020). Data-driven statistical modeling of the emergent behavior of biohybrid microrobots. APL Bioengineering 4, 016104. doi: 10.1063/1.5134926, PMID: 32128471 PMC7049295

[B60] Lee D. Jang E. H. Lee M. Kim S. W. Lee Y. Lee K. T. . (2019a). Unraveling melanin biosynthesis and signaling networks in *Cryptococcus neoformans*. mBio 10, e02267–e02219. doi: 10.1128/mBio.02267-19, PMID: 31575776 PMC6775464

[B61] Lee S. Y. Min J. Lee S. Fitriana H. N. Kim M. S. Park G. W. . (2019b). Bioelectricity generation by *Corynebacterium glutamicum* with redox-hydrogel-modified carbon electrode. Appl. Sci. 9, 4251. doi: 10.3390/app9204251

[B62] Lew R. R. (2011). How does a hypha grow? The biophysics of pressurized growth in fungi. Nat. Rev. Microbiol. 9, 509–518. doi: 10.1038/nrmicro2591, PMID: 21643041

[B63] Li Z. Wu R. Chen K. Gu W. Zhang Y. H. P. Zhu Z. (2023). Enzymatic biofuel cell-powered iontophoretic facial mask for enhanced transdermal drug delivery. Biosensors Bioelectronics 223, 115019. doi: 10.1016/j.bios.2022.115019, PMID: 36563525

[B64] Lin Z. Jiang T. Shang J. (2022). The emerging technology of biohybrid micro-robots: A review. Biodesign Manufacturing 5, 107–132. doi: 10.1007/s42242-021-00135-6

[B65] Lipińska M. B. Maurer C. Cadogan D. Head J. Dade-Robertson M. Paulino-Lima I. G. . (2022). Biological growth as an alternative approach to on and off Earth construction. Front. Built Environ. 8. doi: 10.3389/fbuil.2022.965145

[B66] Liu S. Li X. Wu S. He J. Pang C. Deng Y. . (2014). Fungal pretreatment by *Phanerochaete chrysosporium* for enhancement of biogas production from corn stover silage. Appl. Biochem. Biotechnol. 174, 1907–1918. doi: 10.1007/s12010-014-1185-7, PMID: 25149463

[B67] Liu Y. N. Liu Z. Liu J. Hu Y. Cao B. (2025). Unlocking the potential of *Shewanella* in metabolic engineering: Current status, challenges, and opportunities. Metab. Eng. 89, 1–11. doi: 10.1016/j.ymben.2025.02.002, PMID: 39952391

[B68] Logan B. E. (2008). Microbial fuel cells ( John Wiley & Sons). Available online at: https://www.scirp.org/reference/referencespapers?referenceid=1802126 (Accessed September 18, 2025).

[B69] Lovley D. R. (2012). Electromicrobiology. Annu. Rev. Microbiol. 66, 391–409. doi: 10.1146/annurev-micro-092611-150104, PMID: 22746334

[B70] Lovley D. R. Ueki T. Zhang T. Malvankar N. S. Shrestha P. M. Flanagan K. A. . (2011). Geobacter: The microbe electric’s physiology, ecology, and practical applications. Adv. Microbial Physiol. 59, 1–100. doi: 10.1016/B978-0-12-387661-4.00004-5, PMID: 22114840

[B71] Luke A. K. Burton S. G. (2001). A novel application for *Neurospora crassa*: Progress from batch culture to a membrane bioreactor for the bioremediation of phenols. Enzyme Microbial Technol. 29, 348–356. doi: 10.1016/S0141-0229(01)00390-8

[B72] Mallick N. Bagchi S. K. Koley S. Singh A. K. (2016). Progress and challenges in microalgal biodiesel production. Front. Microbiol. 7. doi: 10.3389/fmicb.2016.01019, PMID: 27446055 PMC4927567

[B73] Marsili E. Baron D. B. Shikhare I. D. Coursolle D. Gralnick J. A. Bond D. R. (2008). *Shewanella* secretes flavins that mediate extracellular electron transfer. Proc. Natl. Acad. Sci. United States America 105, 3968–3973. doi: 10.1073/pnas.0710525105, PMID: 18316736 PMC2268775

[B74] Mazzolai B. Laschi C. (2020). A vision for future bioinspired and biohybrid robots. Sci. Robotics 5, eaba6893. doi: 10.1126/scirobotics.aba6893, PMID: 33022592

[B75] Mehta-Kolte M. G. Bond D. R. (2012). *Geothrix fermentans* secretes two different redox-active compounds to utilize electron acceptors across a wide range of redox potentials. Appl. Environ. Microbiol. 78, 6987–6995. doi: 10.1128/AEM.01460-12, PMID: 22843516 PMC3457516

[B76] Mekuto L. Olowolafe A. V. A. Pandit S. Dyantyi N. Nomngongo P. Huberts R. (2020). Microalgae as a biocathode and feedstock in anode chamber for a self-sustainable microbial fuel cell technology: A review. South Afr. J. Chem. Eng. 31, 7–16. doi: 10.1016/j.sajce.2019.10.002

[B77] Mirza S. S. Abbas A. S. Mansoor D. Morowvat M. H. ur Rehman H. Fatima A. (2025). Assessment of exoelectrogenic potential of microbial consortia of sewage sludge for bioelectricity generation. Int. J. Environ. Sci. Technol. 22, 11645–11654. doi: 10.1007/s13762-024-06318-9

[B78] Mishra A. K. Kim J. Baghdadi H. Johnson B. R. Hodge K. T. Shepherd R. F. (2024). Sensorimotor control of robots mediated by electrophysiological measurements of fungal mycelia. Sci. Robotics 9, eadk8019. doi: 10.1126/scirobotics.adk8019, PMID: 39196952

[B79] Montana-Hoyos C. Daneluzzo M. Tchakerian R. Patel S. V. Morais R. L. (2022). “ Biomimicry and biodesign for innovation in future space colonization,” in Biomimicry for Aerospace: Technologies and Applications (Amsterdam, The Netherlands: Elsevier) 3–39. doi: 10.1016/B978-0-12-821074-1.00012-8

[B80] Mustakeem (2015). Electrode materials for microbial fuel cells: Nanomaterial approach. Materials Renewable Sustain. Energy 4, 1–11. doi: 10.1007/s40243-015-0063-8

[B81] Myers C. R. Nealson K. H. (1988). Bacterial manganese reduction and growth with manganese oxide as the sole electron acceptor. Science 240, 1319–1321. doi: 10.1126/science.240.4857.1319, PMID: 17815852

[B82] Naradasu D. Long X. Okamoto A. Miran W. (2020). “ Bioelectrochemical systems: Principles and applications,” in Bioelectrochemical systems: Vol. 1 Principles and processes. (Singapore: Springer Nature) 1–33. doi: 10.1007/978-981-15-6872-5_1

[B83] Oberdick J. (2025). “ Tiny, soft robot flexes its potential as a life saver,” in Penn State University. (University Park, PA, USA: Materials Research Institute). Available online at: https://www.psu.edu/news/materials-research-institute/story/tiny-soft-robot-flexes-its-potential-life-saver (Accessed September 19, 2025).

[B84] Oda H. Kihara K. Morimoto Y. Takeuchi S. (2021). Cell-based biohybrid sensor device for chemical source direction estimation. Cyborg Bionic Syst. 2021, 8907148. doi: 10.34133/2021/8907148, PMID: 36285129 PMC9494699

[B85] Oliveira V. B. Simões M. Melo L. F. Pinto A. M. F. R. (2013). Overview on the developments of microbial fuel cells. Biochem. Eng. J. 73, 53–64. doi: 10.1016/j.bej.2013.03.006

[B86] Osman M. E. Khattab O. H. Elnasr A. A. Basset A. S. (2018). Bioelectricity generation and dye decolorization by *Aspergillus Niger* and *Trichoderma harzianum*. doi: 10.4172/2155-6199.1000446

[B87] Ostergaard S. Olsson L. Nielsen J. (2000). Metabolic engineering of *Saccharomyces cerevisiae*. Microbiol. Mol. Biol. Rev. 64, 34–50. doi: 10.1128/MMBR.64.1.34-50.2000, PMID: 10704473 PMC98985

[B88] Ottoni C. do Valle Trotta C. Martins G. Matos J. Maiorano A. E. Brito A. G. . (2023). *In situ Trametes versicolor* laccase biocathode performance assessment in dual-chamber microbial fuel cells. Bioenergy Res. 16, 2616–2624. doi: 10.1007/s12155-023-10594-7

[B89] Pal J. Kumar N. (2017). Comparative study on single and double chambered microbial fuel cell. Available online at: http://www.sedIndia.org (Accessed September 23, 2025).

[B90] Pandit S. Nayak B. K. Das D. (2012). Microbial carbon capture cell using cyanobacteria for simultaneous power generation, carbon dioxide sequestration and wastewater treatment. Bioresource Technol. 107, 97–102. doi: 10.1016/j.biortech.2011.12.067, PMID: 22221988

[B91] Pant D. Singh A. Van Bogaert G. Olsen S. I. Singh Nigam P. Diels L. . (2012). Bioelectrochemical systems (BES) for sustainable energy production and product recovery from organic wastes and industrial wastewaters. RSC Adv. 2, 1248–1263. doi: 10.1039/C1RA00839K

[B92] Parihar P. S. Keshavkant S. Jadhav S. K. (2022). Electrogenic potential of *Enterococcus faecalis* DWW1 isolated from the anodic biofilm of a dairy wastewater fed dual chambered microbial fuel cell. J. Water Process Eng. 45, 102503. doi: 10.1016/j.jwpe.2021.102503

[B93] Pham M. T. Tran T. D. Zayabaatar E. (2022). *Leuconostoc mesenteroides* utilizes glucose fermentation to produce electricity and ameliorates high-fat diet-induced abdominal fat mass. Arch. Microbiol. 204, 1–11. doi: 10.1007/s00203-022-03281-2, PMID: 36241916

[B94] Phillips N. Gandia A. Adamatzky A. (2023). Electrical response of fungi to changing moisture content. Fungal Biol. Biotechnol. 10. doi: 10.1186/s40694-023-00155-0, PMID: 37013653 PMC10069029

[B95] Piñeros V. J. Reveles-Espinoza A. M. Monroy J. A. (2024). From remote sensing to artificial intelligence in coral reef monitoring. Machines 12, 693. doi: 10.3390/machines12100693

[B96] Pisciotta J. M. Blessing S. (2022). “ Microbial bioelectricity generation and product electrosynthesis,” in Industrial microbiology and biotechnology. (Singapore: Springer Nature) 505–554. doi: 10.1007/978-981-16-5214-1_18

[B97] Potter M. C. (1911). Electrical effects accompanying the decomposition of organic compounds. Proc. R. Soc. London. Ser. B 84, 260–276. doi: 10.1098/rspb.1911.0073

[B98] Rahimnejad M. Najafpour G. D. Ghoreyshi A. A. Talebnia F. Premier G. C. Bakeri G. . (2012). Thionine increases electricity generation from microbial fuel cell using *Saccharomyces cerevisiae* and exoelectrogenic mixed culture. J. Microbiol. 50, 575–580. doi: 10.1007/s12275-012-2135-0, PMID: 22923104

[B99] Ramya M. Senthil Kumar P. (2022). A review on recent advancements in bioenergy production using microbial fuel cells. Chemosphere 288, 132512. doi: 10.1016/j.chemosphere.2021.132512, PMID: 34634275

[B100] Reddy C. N. Kakarla R. Min B. (2019). “ Algal biocathodes,” in Biomass, biofuels, biochemicals: Microbial electrochemical technology: Sustainable platform for fuels, chemicals and remediation. (Amsterdam, The Netherlands: Elsevier) 525–547. doi: 10.1016/B978-0-444-64052-9.00021-2

[B101] Reguera G. Kashefi K. (2019). The electrifying physiology of *Geobacter* bacteria, 30 years on. Adv. Microbial Physiol. 74, 1–96. doi: 10.1016/bs.ampbs.2019.02.007, PMID: 31126529

[B102] Reyes C. Fivaz E. Sajó Z. Schneider A. Siqueira G. Ribera J. . (2024). 3D printed cellulose-based fungal battery. ACS Sustain. Chem. Eng. 12, 16001–16011. doi: 10.1021/acssuschemeng.4c07123

[B103] Reyes C. Qian F. Zhang A. Bondarev S. Welch A. Thelen M. P. . (2012). Characterization of axial and proximal histidine mutations of the decaheme cytochrome mtra from shewanella sp. Strain ANA-3 and implications for the electron transport system. J. Bacteriol 194, 5840–5847. doi: 10.1128/jb.00890-12, PMID: 22923588 PMC3486075

[B104] Riaz A. Sarker M. R. Saad M. H. M. Mohamed R. (2021). Review on comparison of different energy storage technologies used in micro-energy harvesting, WSNs, low-cost microelectronic devices: Challenges and recommendations. Sensors 21, 5041. doi: 10.3390/s21155041, PMID: 34372278 PMC8428241

[B105] Rojas-Flores S. de la Cruz-Noriega M. Otiniano N. M. Cabanillas-Chirinos L. (2024). Sustainable use of the fungus Aspergillus sp. to simultaneously generate electricity and reduce plastic through microbial fuel cells. Sustainability 16, 7413. doi: 10.3390/su16177413

[B106] Rotaru A. E. Shrestha P. M. Liu F. Markovaite B. Chen S. Nevin K. P. . (2014). Direct interspecies electron transfer between *Geobacter metallireducens* and *Methanosarcina barkeri*. Appl. Environ. Microbiol. 80, 4599–4605. doi: 10.1128/AEM.00895-14, PMID: 24837373 PMC4148795

[B107] Rus D. Tolley M. T. (2015). Design, fabrication and control of soft robots. Nature 521, 467–475. doi: 10.1038/nature14543, PMID: 26017446

[B108] Ryu J. Choi S. (2021). Bioelectricity production from sweat-activated germination of bacterial endospores. Biosensors Bioelectronics 186, 113293. doi: 10.1016/j.bios.2021.113293, PMID: 33964796

[B109] Saratale R. G. Kuppam C. Mudhoo A. Saratale G. D. Periyasamy S. Zhen G. . (2017). Bioelectrochemical systems using microalgae – A concise research update. Chemosphere 177, 35–43. doi: 10.1016/j.chemosphere.2017.02.132, PMID: 28284115

[B110] Sari Sekar Ningrum A. Zulaika A. Handini W. Prasetyo D. D. (2023). Bioelectricity from dual chamber microbial fuel cell (MFC) using *Aspergillus Niger* with sugarcane bagasse substrate. Jurnal Penelitian Pendidikan IPA. doi: 10.29303/jppipa.v9iSpecialIssue.6437

[B111] Sarma H. Bhattacharyya P. N. Jadhav D. A. Pawar P. Thakare M. Pandit S. . (2021). Fungal-mediated electrochemical system: Prospects, applications and challenges. Curr. Res. Microbial Sci. 2, 100041. doi: 10.1016/j.crmicr.2021.100041, PMID: 34841332 PMC8610361

[B112] Sarma R. Kakati B. K. (2022). “ Modern challenges and future perspective of microbial fuel cells,” in Bioelectrochemical systems: Principles and applications. (Singapore: Springer Nature) 429–446. doi: 10.1007/978-981-19-2681-5_19

[B113] Sawa M. Fantuzzi A. Bombelli P. Howe C. J. Hellgardt K. Nixon P. J. (2017). Electricity generation from digitally printed cyanobacteria. Nat. Commun. 8, 1–10. doi: 10.1038/s41467-017-01084-4, PMID: 29109396 PMC5673893

[B114] Sayed E. T. Abdelkareem M. A. (2017). Yeast as a biocatalyst in microbial fuel cell. Old Yeasts – New Questions 317, 41–65.

[B115] Schaetzle O. Barrière F. Baronian K. (2008). Bacteria and yeasts as catalysts in microbial fuel cells: Electron transfer from micro-organisms to electrodes for green electricity. Energy Environ. Sci. 1, 607–620. doi: 10.1039/b810642h

[B116] Sharma A. Rashid M. Chauhan P. Kaur S. Kaur A. (2024). *In vitro* antibacterial and anti-biofilm potential of an endophytic *Schizophyllum commune*. AMB Express 14, 1–14. doi: 10.1186/s13568-024-01663-x, PMID: 38245627 PMC10799838

[B117] Shrivastava A. Sharma R. K. (2023). Conversion of lignocellulosic biomass: Production of bioethanol and bioelectricity using wheat straw hydrolysate in electrochemical bioreactor. Heliyon 9, e12951. doi: 10.1016/j.heliyon.2023.e12951, PMID: 36711303 PMC9873701

[B118] Shukla M. Kumar S. (2018). Algal growth in photosynthetic algal microbial fuel cell and its subsequent utilization for biofuels. Renewable Sustain. Energy Rev. 82, 402–414. doi: 10.1016/j.rser.2017.09.067

[B119] Sisler F. D. (1961). Electrical energy from biochemical fuel. New Scientist 12, 110e1. Available online at: https://scholar.google.com/scholar?hl=en&as_sdt=0%2C5&q=Sisler+FD.+Electrical+energy+from+biochemical+fuel+cells.+NewSci+1961%3B12%3A110e1 (Accessed September 21, 2025).

[B120] Sisler F. D. (1962). Electrical energy from microbiological processes. Available online at: https://www.jstor.org/stable/24535009 (Accessed September 24, 2025).

[B121] Sleutels T. H. J. A. Ter Heijne A. Buisman C. J. N. Hamelers H. V. M. (2012). Bioelectrochemical systems: An outlook for practical applications. ChemSusChem 5, 1012–1019. doi: 10.1002/cssc.201100732, PMID: 22674691

[B122] Sreelekshmy B. R. (2020). Exploration of electrochemically active bacterial strains for microbial fuel cells: An innovation in bioelectricity generation. J. Pure Appl. Microbiol. 14, 103–122. doi: 10.22207/JPAM.14.1.12

[B123] Strik D. P. B. T. B. Timmers R. A. Helder M. Steinbusch K. J. J. Hamelers H. V. M. Buisman C. J. N. (2011). Microbial solar cells: Applying photosynthetic and electrochemically active organisms. Trends Biotechnol. 29, 41–49. doi: 10.1016/j.tibtech.2010.10.001, PMID: 21067833

[B124] Taha M. F. Mao H. Zhang Z. Elmasry G. Awad M. A. Abdalla A. . (2025). Emerging technologies for precision crop management towards Agriculture 5.0: A comprehensive overview. Agriculture 15, 582. doi: 10.3390/agriculture15060582

[B125] Tanaka K. Tamamushi R. Ogawa T. (1985). Bioelectrochemical fuel-cells operated by the cyanobacterium *Anabaena variabilis*. J. Chem. Technol. Biotechnol. 35, 191–197. doi: 10.1002/jctb.280350304

[B126] Tanguay-Rioux F. Nwanebu E. Thadani M. Tartakovsky B. (2023). On-line current control for continuous conversion of CO2 to CH4 in a microbial electrosynthesis cell. Biochem. Eng. J. 197, 108965. doi: 10.1016/j.bej.2023.108965

[B127] Tejada J. C. Toro-Ossaba A. López-Gonzalez A. Hernandez-Martinez E. G. Sanin-Villa D. (2025). A review of multi-robot systems and soft robotics: Challenges and opportunities. Sensors 25, 1353. doi: 10.3390/s25051353, PMID: 40096190 PMC11902459

[B128] Thapa B. S. Kim T. Pandit S. Song Y. E. Afsharian Y. P. Rahimnejad M. . (2022). Overview of electroactive microorganisms and electron transfer mechanisms in microbial electrochemistry. Bioresource Technol. 347, 126579. doi: 10.1016/j.biortech.2021.126579, PMID: 34921921

[B129] Umar A. Mubeen M. Ali I. Iftikhar Y. Sohail M. A. Sajid A. . (2023). Harnessing fungal bio-electricity: A promising path to a cleaner environment. Front. Microbiol. 14. doi: 10.3389/fmicb.2023.1291904/full, PMID: 38352061 PMC10861785

[B130] Vargas M. Malvankar N. S. Tremblay P. Leang C. Smith J. A. Patel P. . (2013). Aromatic amino acids required for pili conductivity and long range extracellular electron transport in *Geobacter sulfurreducens*. mBio 4, e00105–e00113. doi: 10.1128/mBio.00105-13, PMID: 23481602 PMC3604773

[B131] Vašatko H. Gosch L. Jauk J. Stavric M. (2022). Basic research of material properties of mycelium-based composites. Biomimetics 7, 51. doi: 10.3390/biomimetics7020051/s1, PMID: 35645178 PMC9150003

[B132] Vishwanathan A. S. (2021). Microbial fuel cells: A comprehensive review for beginners. 3 Biotech. 11, 1–14. doi: 10.1007/s13205-021-02802-y, PMID: 33968591 PMC8088421

[B133] Wang L. Pang Q. Peng F. Zhang A. Zhou Y. Lian J. . (2020). Response characteristics of nitrifying bacteria and archaea community involved in nitrogen removal and bioelectricity generation in integrated tidal flow constructed wetland-microbial fuel cell. Front. Microbiol. 11. doi: 10.3389/fmicb.2020.01385/bibtex, PMID: 32655535 PMC7324634

[B134] Wang X. Xia K. Yang X. Tang C. (2019). Growth strategy of microbes on mixed carbon sources. Nat. Commun. 10, 1–7. doi: 10.1038/s41467-019-09261-3, PMID: 30894528 PMC6427025

[B135] Webster-Wood V. A. Guix M. Xu N. W. Behkam B. Sato H. Sarkar D. . (2022). Biohybrid robots: Recent progress, challenges, and perspectives. Bioinspiration Biomimetics 18, 015001. doi: 10.1088/1748-3190/ac9c3b, PMID: 36265472

[B136] Wilhelmsen C. O. Kristensen S. B. Nolte O. Volodin I. A. Christiansen J. V. Isbrandt T. . (2023). Demonstrating the use of a fungal synthesized quinone in a redox flow battery. Batteries Supercaps 6, e202200365. doi: 10.1002/batt.202200365

[B137] Wu Z. W. Zhao X. F. Quan C. X. Liu X. C. Tao X. Y. Li Y. J. . (2025). Structure–function insights of natural *Ganoderma* polysaccharides: Advances in biosynthesis and functional food applications. Natural Products Bioprospecting 15, 1–33. doi: 10.1007/s13659-025-00496-w/tables/3, PMID: 40035898 PMC11880470

[B138] Xie R. Wang S. Wang K. Wang M. Chen B. Wang Z. . (2022). Improved energy efficiency in microbial fuel cells by bioethanol and electricity co-generation. Biotechnol. Biofuels Bioproducts 15, 1–14. doi: 10.1186/s13068-022-02180-4/tables/3, PMID: 35978352 PMC9382818

[B139] Xu C. Poon K. Choi M. M. F. Wang R. (2015). Using live algae at the anode of a microbial fuel cell to generate electricity. Environ. Sci. pollut. Res. 22, 15621–15635. doi: 10.1007/s11356-015-4744-8/metrics, PMID: 26018284

[B140] Yin X. Wang F. Ge M. Zhang F. Liang G. (2025). *Pseudomonas aeruginosa* promoted microbial fuel cells for cytidine acid production wastewater treatment. Sci. Rep. 15, 1–14. doi: 10.1038/s41598-025-90361-0, PMID: 40065049 PMC11894049

[B141] Yu L. He D. Zhang E. He Q. Li J. Ren J. Z. . (2021). Electricity from anaerobic methane oxidation by a single methanogenic archaeon *Methanosarcina barkeri*. Chem. Eng. J. 405, 126691. doi: 10.1016/j.cej.2020.126691

[B142] Yuan Y. Zhou S. Xu N. Zhuang L. (2011). Electrochemical characterization of anodic biofilms enriched with glucose and acetate in single-chamber microbial fuel cells. Colloids Surfaces B: Biointerfaces 82, 641–646. doi: 10.1016/j.colsurfb.2010.10.015, PMID: 21050727

[B143] Zarepour A. Khosravi A. Iravani S. Zarrabi A. (2024). Biohybrid micro/nanorobots: Pioneering the next generation of medical technology. Advanced Healthcare Materials 13, 2402102. doi: 10.1002/adhm.202402102, PMID: 39373299 PMC11650542

[B144] Zhang S. Yang C. Jiang Y. Li P. Xia C. (2023). A robust fluorine-containing ceramic cathode for direct CO2 electrolysis in solid oxide electrolysis cells. J. Energy Chem. 77, 300–309. doi: 10.1016/j.jechem.2022.10.021

[B145] Zhang L. Zhang Y. Liu Y. Wang S. Lee C. K. Huang Y. . (2024). High-power density redox-mediated Shewanella microbial flow fuel cells. Nat. Commun. 15, 8302. doi: 10.1038/s41467-024-52498-w, PMID: 39333111 PMC11448506

[B146] Zhao J. Li F. Cao Y. Zhang X. Chen T. Song H. . (2021). Microbial extracellular electron transfer and strategies for engineering electroactive microorganisms. Biotechnol. Adv. 53, 107682. doi: 10.1016/j.bioteChadv.2020.107682, PMID: 33326817

[B147] Zou P. Li P. Liu J. Cao P. Luan Q. (2022). Direct current exerts electricidal and bioelectric effects on *Porphyromonas gingivalis* biofilms partially via promoting oxidative stress and antibiotic transport. J. Microbiol. 60, 70–78. doi: 10.1007/s12275-022-1238-5/metrics, PMID: 34826101

